# Structural insights into the elevator-like mechanism of the sodium/citrate symporter CitS

**DOI:** 10.1038/s41598-017-02794-x

**Published:** 2017-05-31

**Authors:** Ji Won Kim, Subin Kim, Songwon Kim, Haerim Lee, Jie-Oh Lee, Mi Sun Jin

**Affiliations:** 1 Department of Chemistry, KAIST, 291 Daehak-ro, Yuseong-gu, Daejeon 34141 Republic of Korea; 2 School of Life Sciences, GIST, 123 Cheomdan-gwagiro, Buk-gu, Gwangju 61005 Republic of Korea

## Abstract

The sodium-dependent citrate transporter of *Klebsiella pneumoniae* (*Kp*CitS) belongs to the 2-hydroxycarboxylate transporter (2-HCT) family and allows the cell to use citrate as sole carbon and energy source in anaerobic conditions. Here we present crystal structures of *Kp*CitS in citrate-bound outward-facing, citrate-bound asymmetric, and citrate-free inward-facing state. The structures reveal that the *Kp*CitS dimerization domain remains stationary throughout the transport cycle due to a hydrogen bond network as well as extensive hydrophobic interactions. In contrast, its transport domain undergoes a ~35° rigid-body rotation and a ~17 Å translocation perpendicular to the membrane to expose the substrate-binding site alternately to either side of the membrane. Furthermore, homology models of two other 2-HCT proteins based on the *Kp*CitS structure offer structural insights into their differences in substrate specificity at a molecular level. On the basis of our results and previous biochemical data, we propose that the activity of the 2-HCT CitS involves an elevator-like movement in which the transport domain itself traverses the lipid bilayer, carrying the substrate into the cell in a sodium-dependent manner.

## Introduction

The 2-HCT family of transporters generally translocate molecules with a 2-hydroxycarboxylate motif (HO-CR_1_R_2_-COO^−^), such as citrate, malate and lactate across the plasma membrane, and activity is tightly coupled to energy provided by a sodium or proton gradient as a form of secondary active transport^1, 2^. *Kp*CitS is the best-studied model system, and has been functionally isolated in detergent and characterized in a reconstituted state^3–5^. It plays a key role in citrate uptake ultimately leading to the production of ATP in anaerobic fermentative process^2, 6–8^. Analysis of hydropathy profiles of *Kp*CitS and rich biochemical data suggested that it consists of 11 transmembrane helices with two putative reentrant loops, now referred to as helical hairpins (HP)^9–12^. Single-molecule fluorescence spectroscopy has provided evidence that it functions as a homodimer^13^. Mutational studies have shown that R428, which is strictly conserved in transporters of the 2-HCT family, is critical for interaction with one of the carboxylate groups of citrate^5, 14, 15^. Analysis of data from kinetics experiments demonstrated that *Kp*CitS transports citrate followed by binding of sodium ion^4, 16^. However, there are conflicting data regarding exact stoichiometry^7, 16–19^. The structure of *Kp*CitS has been studied extensively by electron crystallography, providing a glimpse of its global structure and a clue to the substrate-induced conformational changes^20, 21^. The crystal structure of a homologous symporter from *Salmonella enterica* (*Se*CitS) recently revealed that it forms an asymmetric dimer, and that each protomer embeds a substrate translocation pathway at the interface between the transport and the dimerization domains^22^. That structure provided the first high resolution view of a member of the 2-HCT family; however, many details in the transport cycle remained unanswered.

## Results

### Structure determination and overall architecture

In order to understand the mechanism of the 2-HCT family in more detail, we sought to determine the *Kp*CitS structure by X-ray crystallography in different functional states. For this purpose, membranes of bacteria expressing *Kp*CitS were solubilized with n-dodecyl-β-D-maltopyranoside (DDM), and the purified protein was crystallized in a sodium-containing buffer with and without citrate. The structures were determined by molecular replacement using separate protomers of the *Se*CitS structure as search probes (Supplementary Tables 1 and 2)^22^. The final models showed that two crystal forms of *Kp*CitS were obtained in the citrate-containing droplet. One crystal form (form 1), refined at 4.0 Å resolution, contains an outward-facing *Kp*CitS homodimer (Supplementary Fig. 1). In contrast, the other crystal form (form 2), refined to 3.8 Å resolution, contains two pairs of *Kp*CitS dimers in an asymmetric unit; one has an outward-facing conformation, and the other an asymmetric conformation. Citrate-free *Kp*CitS yielded a crystal diffracting to 3.6 Å resolution (form 3) with two inward-facing *Kp*CitS dimers in an asymmetric unit. The dimers are practically identical, with Cα rms differences below 0.3 Å, suggesting that this state is structurally stable in the crystallization conditions. We also tried solubilizing bacterial membranes with n-decyl-β-D-maltopyranoside as used in the structure determination of *Se*CitS^22^. Interestingly, this method only produced a crystal of an asymmetric *Kp*CitS homodimer in the presence of citrate (form 4), and it was refined at a resolution of 3.5 Å. For the sake of the accuracy, we selected the structure of the highest resolution dataset from each of the three major conformational states for analysis; thus, the outward-facing, inward-facing, and asymmetric conformations of crystal forms 2, 3, and 4, respectively, are shown throughout this article (Supplementary Figs 2, 3 and 4).

**Figure 1:**
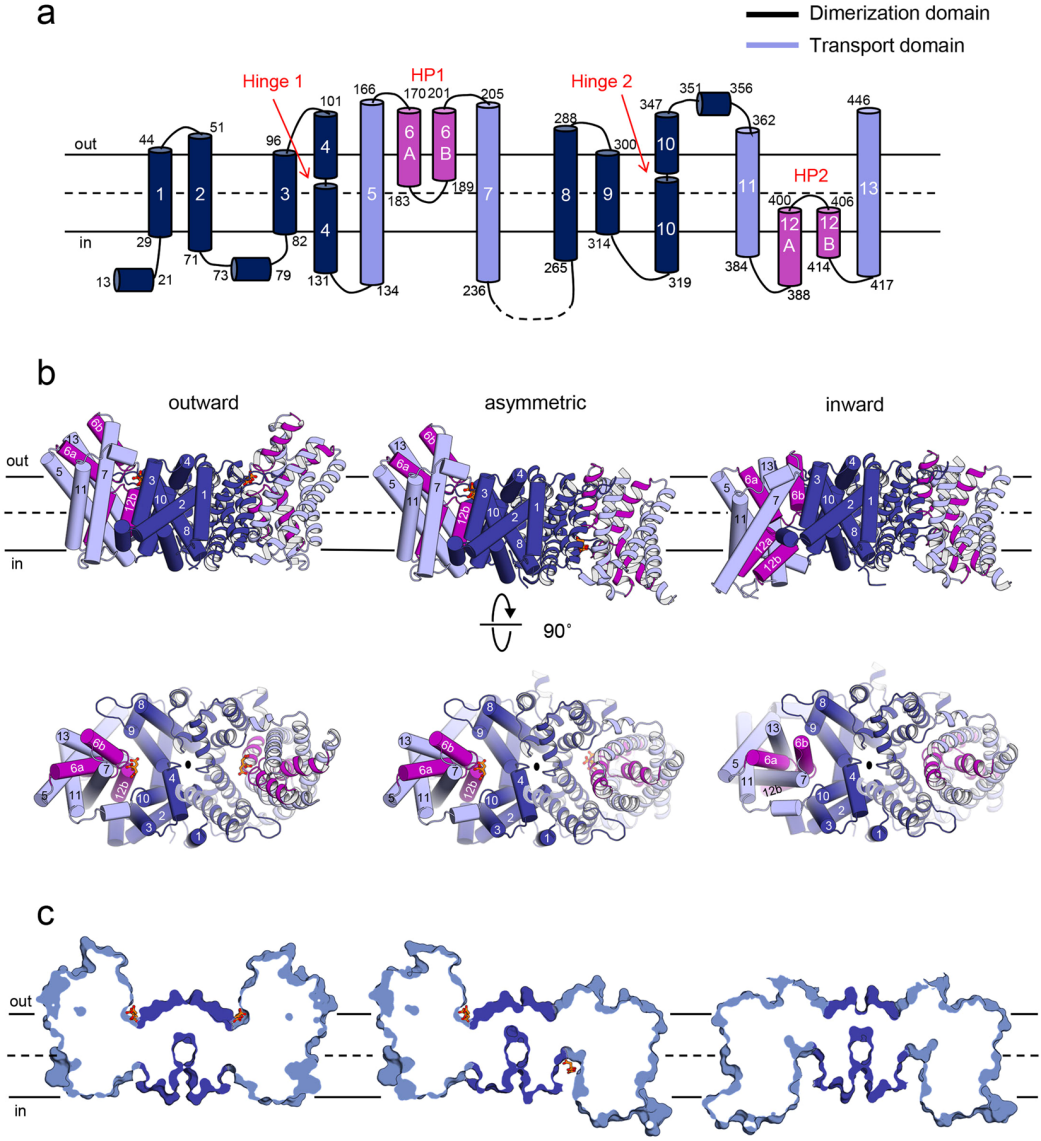
The three conformational states of the *Kp*CitS dimer. (**a**) Schematic representation of the *Kp*CitS protomer. The helices belonging to the dimerization and the transport domains are in dark and pale blue, respectively. Two helical hairpins of the transport domain are highlighted in purple. Residues in the loop connecting TM 7 and TM 8 are not included in the crystal structures due to their low electron density, and are drawn with a dashed line. Protein secondary structures were assigned using the STRIDE^53^. (**b**) The homodimeric *Kp*CitS structure in different functional states viewed from the membrane plane (top) and from the periplasm (bottom). Citrate is shown as an orange ball-and-stick model. The black oval is a pseudo 2-fold axis, perpendicular to the membrane. (**c**) Surface slab views of (**a**).

Our crystal structures reveal that the *Kp*CitS protomer is formed by homologous N-terminal and C-terminal halves with inverted topology, each containing six transmembrane helices (TM 2–5, 7 and TM 8–11, 13) and one helical hairpin (TM 6a/b (or HP1) and TM 12a/b (or HP2)) (Fig. 1a). TM 1 with the N-terminus toward cytoplasm is not involved in either of the two halves. *Kp*CitS protomer can be divided into two domains; a central dimerization domain (TM 1–4 and 8–10) critical for formation of the dimeric structure, and a peripheral transport domain (TM 5–7 and 11–13) containing residues needed for interaction with citrate and sodium ions. The outward- and inward-facing structures contain a pseudo two-fold symmetry at the center (Fig. 1b). Each *Kp*CitS protomer binds one molecule of citrate in the outward-facing and asymmetric conformations, indicating that the functional unit for citrate uptake and transport is a monomer (Fig. 1b,c). As expected from the 92% sequence identity, the CitS structures obtained from *Klebsiella pneumoniae* and *Salmonella enterica* are highly homologous. The Cα rms differences of the backbone atoms are ~1.1 Å for the outward-facing, and ~0.3 Å for the inward-facing protomers, suggesting that they have very similar overall structures and that their mechanism of substrate transport is conserved (Supplementary Fig. 5). Throughout this article, single apostrophes are used for the residues of one protomer to differentiate them from those of the other protomer.

### The dimerization domains of *Kp*CitS in the transport cycle

**Figure 2:**
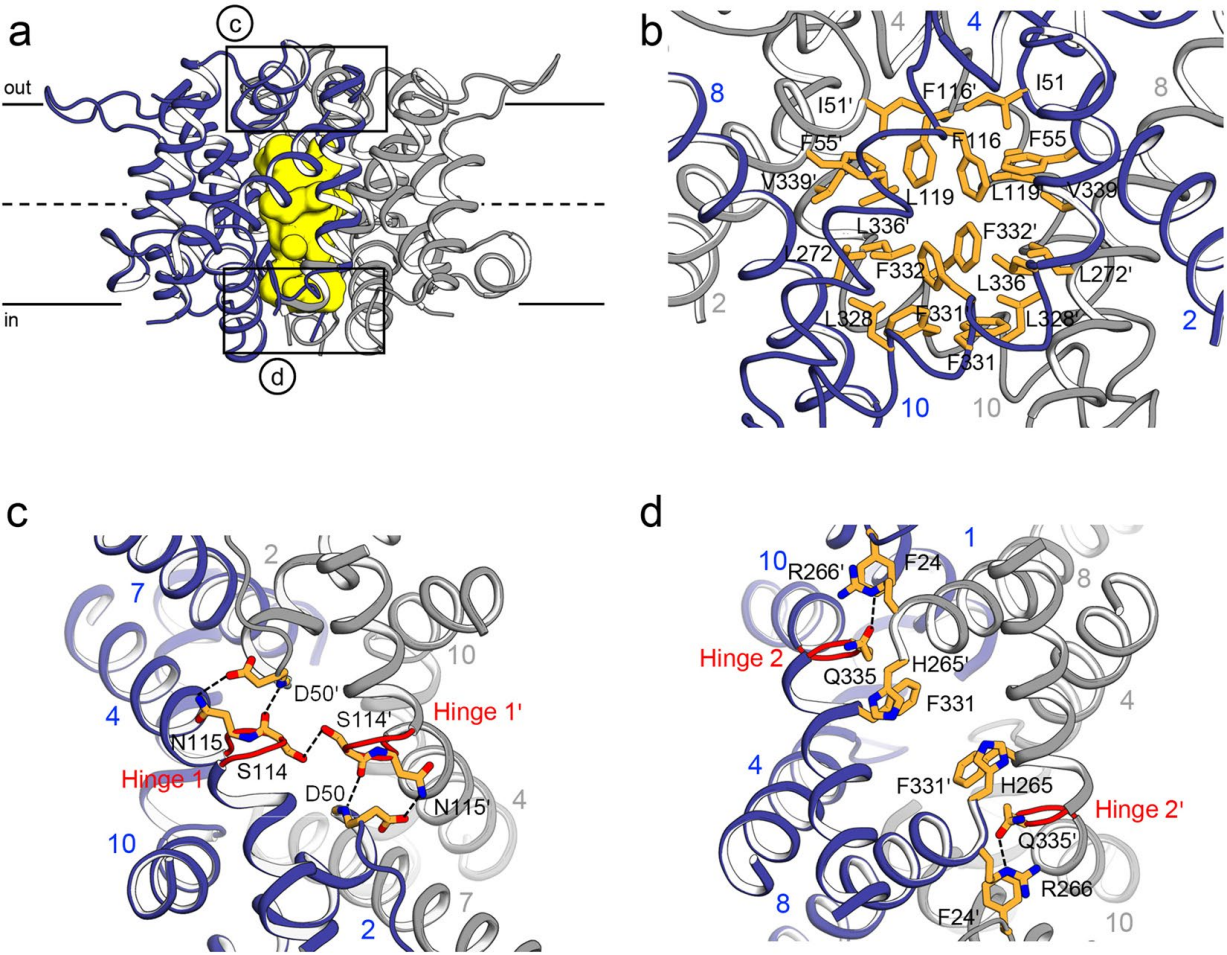
The interface of the *Kp*CitS homodimer. (**a**) Ribbon representation of the dimerization domains. The hydrophobic cavity is highlighted in yellow. The two areas enlarged in (**c**,**d**) are boxed. The dimerization domains in one dimer are shown in blue and grey, respectively. (**b**) The shape of the central cavity in the dimerization interface, and the residues lining the cavity. The view is rotated by 90 degrees along the vertical axis from that of (**a**). (**c**) Residues involved in stabilizing the dimer interface on the periplasmic side of *Kp*CitS. (**d**) Residues involved in dimer stabilization on the cytoplasmic side of *Kp*CitS. The hinge 1 and 2 regions on TM 4 and TM 10, respectively, are highlighted in red.

The crystal structures in the three functional states show that the dimerization domains remain fixed throughout the transport cycle due to extensive hydrophobic interactions (Fig. 1 and Supplementary Fig. 6). The dimer interface has an approximate area of 2,700 Å^2^, and contains a large hydrophobic cavity with a volume of 900 Å^3^ (Fig. 2a). The internal cavity is semi-circular and is completely lined with hydrophobic residues, mainly from helices TM 2, 4, 8 and 10 (Fig. 2b). One detergent molecule of n-octyl-β-D-glucopyranoside (β-OG), used as additive in protein crystallization, fills the central cavity at the level of the membrane’s inner leaflet (Supplementary Fig. 7). Consistent with the hydrophobic nature of the cavity, the hydrophilic head group of β-OG is exposed to the cytoplasm, and the non-polar carbon tail is inserted into the cavity in an extended conformation, with a structure that is nearly identical to that of the lipid chain within the dimeric interface of *Se*CitS^22^. Since the cavity is large enough to bind the long lipid acyl chains, *Kp*CitS within a native membrane environment would permit the entry of a lipid molecule, which may have some effect on the stability and activity of *Kp*CitS by virtue of hydrophobic interactions with residues of the dimer interface^23^.

Another notable feature of the *Kp*CitS dimer interface is that hinge-like structures (Hinge 1 and 2) surround the entry of the cavity on both sides of the membrane (Figs 1a and 2c,d). Together with cation-π (R266-F24′) and π-π (F331-H265′) interactions, these hinges create an intermolecular hydrogen bonding network (D50-N115′, S114-S114′, the backbone nitrogen of D50 and the backbone oxygen of S114′, R266-Q335′) that further stabilizes the dimeric structure (Supplementary Discussion and Supplementary Fig. 8). Q335 on Hinge 2 also plays a role in fixing the conformation of R266′ for effective cation-π interaction with F24 (Fig. 2d).

### The substrate binding and its transport

**Figure 3:**
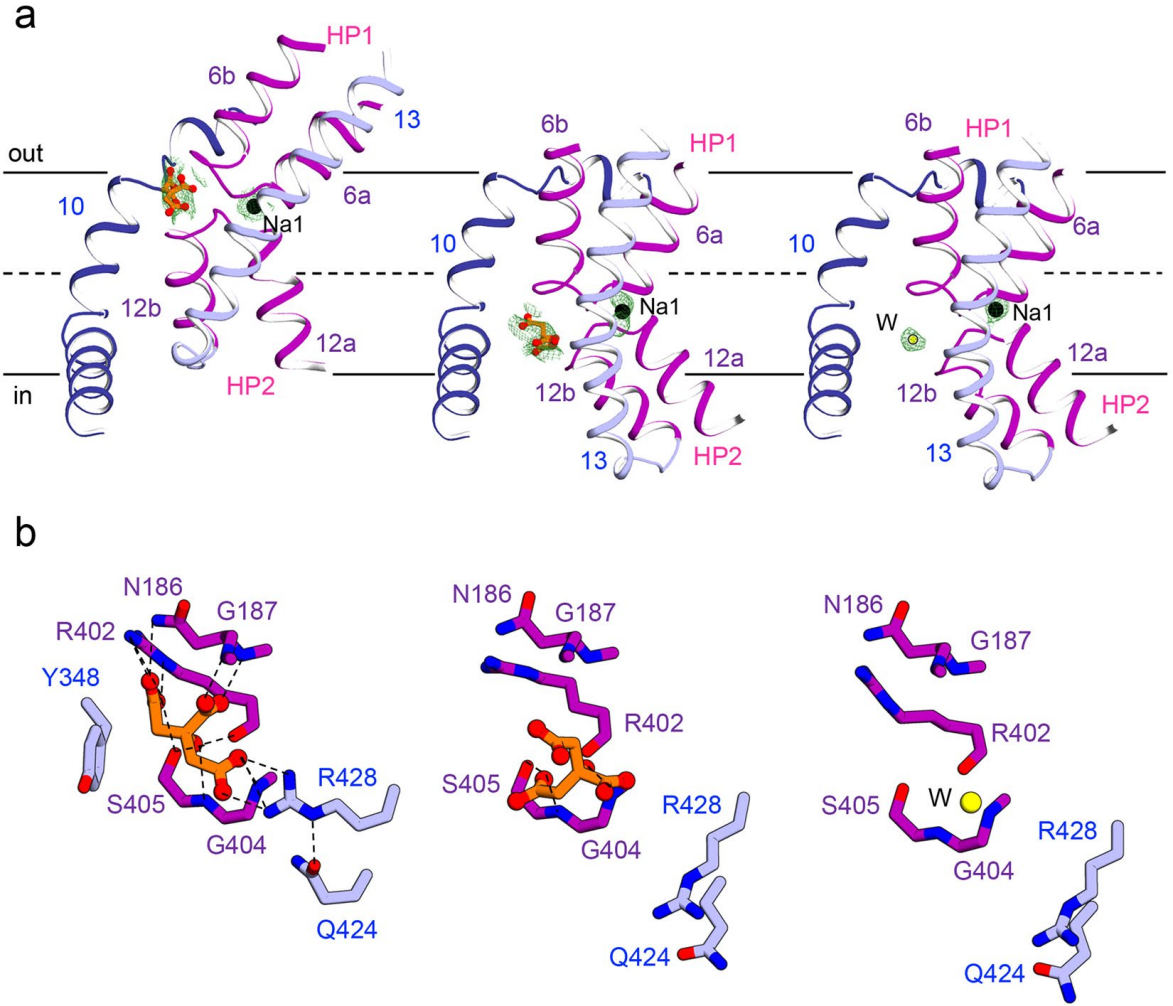
The substrate binding site of *Kp*CitS. (**a**) The helical hairpins, HP1 and HP2, of the transport domain in the citrate-bound outward-facing (left), citrate-bound inward-facing (middle), and citrate-free inward-facing^44^ states are represented in purple. Citrate is represented by an orange ball-and-stick model, sodium ions (Na1) by black spheres, and water (W) by a yellow sphere. The simulated annealing omit maps superimposed on the refined citrate, sodium ion and water were contoured at the 3σ levels. (**b**) Close-up view of the substrate binding site in the citrate-bound outward-facing (left), citrate-bound inward-facing (middle), and citrate-free inward-facing^44^ conformations. The hydrogen bonds and ionic interactions are shown by dashed lines.

The transport domain of *Kp*CitS undergoes a large conformational change associated with substrate binding (Fig. 1 and Supplementary Fig. 6). The most obvious changes are concentrated on the two helical hairpins HP1 and HP2 arranged vertical to the membrane (Fig. 3a). In the outward-facing state, the hairpin loops are located close to the membrane/periplasm interface, which appears to favor the entry of external substrate and ions (Fig. 3a, left). This is consistent with mutational and cross-linking data showing that the hairpin loops, which contain conserved GGxG motives, are positioned close together to form the binding sites for the citrate and sodium ions^24, 25^. In the inward-facing state, whether or not citrate binding, the hairpins slide downwards relative to the dimerization domain as a result of an approximately 35° rigid-body rotation and 17 Å translocation perpendicular to the membrane; this opens the substrate transport pathway to the cytoplasm as far as the inner leaflet of the membrane (Figs 1c and 3a, middle and right).

The substrate transport pathway of *Kp*CitS is located between the transport and dimerization domains (Figs 1b,c and 3a). In the outward-facing state the citrate is firmly fixed by essential hydrogen bonds and ionic interactions as follows (Fig. 3b, left and Supplementary Fig. 9a): (i) the hydroxyl group of the 2-hydroxycarboxylate motif of citrate forms hydrogen bonds with the hydroxyl side chain and the backbone nitrogen of S405, and with the backbone oxygen of R402, (ii) the carboxylate group of the 2-hydroxycarboxylate motif forms hydrogen bonds with the backbone nitrogens of N186 and G187, (iii) the 1-carboxylate group of the citrate makes ionic and hydrogen bonds with the side chain of R428 and the backbone nitrogen of G404, and (iv) the 3-carboxylate group of citrate makes hydrogen bonds with the side chains of N186 and R402. Y348 on TM 10 also participates in the coordination of citrate via an anion-π interaction with the 3-carboxylate group^26–28^. The highly conserved Q424 is not directly involved in the coordination, but contributes to orienting R428 to permit it to form strong hydrogen bonds with the 1-carboxylate group of citrate. In the outward-facing state the bound citrate is partially exposed to the periplasmic space. However, it is unlikely that it can escape backwards because it is secured by strong hydrogen and electrostatic interactions, as mentioned above. This is different from the crystal structures of other secondary active transporters such as GltPh, TtNapA, SLC26_Dg, PaNhaP, MjNhaP1, BetP and UapA, in which the substrate is completely occluded from the aqueous phase^29–38^.

In the citrate-bound inward-facing state, the backbone structure of the substrate binding site hardly changes compared to that in the outward-facing state. However, the critical side chains of the residues coordinating the citrate move substantially (Fig. 3b, left and middle). The side chain of Q424 rotates by 55°, which results in a conformational change of R428, and weakens the ionic interaction with the 1-carboxylate group of citrate. Furthermore, the entire transport domain moves downwards relative to the dimerization domain, so that Y348 on TM10 completely loses the ability to coordinate the 3-carboxylate group of citrate. Consequently, the citrate is weakly attached only to the side chain and the backbone nitrogen of S405 and the backbone nitrogen of G404 (Fig. 3b, middle and Supplementary Fig. 9b). They are consistent with the *Kp*CitS proteoliposome data showing that citrate transport occurs in an asymmetric conformation in which one binding site has a high and the other a low affinity for substrate^4^. In the citrate-free inward-facing state, we observed that one water molecule takes the place of a citrate (Fig. 3a,b, right). Since the substrate binding site is continuous with the cytoplasm, water molecules are able to move deep into the membrane inner leaflet. In addition to the changes in amino acid conformation as described above, polar water molecules also seem to facilitate release of the substrate into the cell by breaking the electrostatic interactions between substrate and transporter. This idea is supported by the fact that the bound citrate in the inward-facing protomer is only partially hydrated in the *Se*CitS structure^22^.

### Substrate selectivity of the 2-HCT family

**Figure 4:**
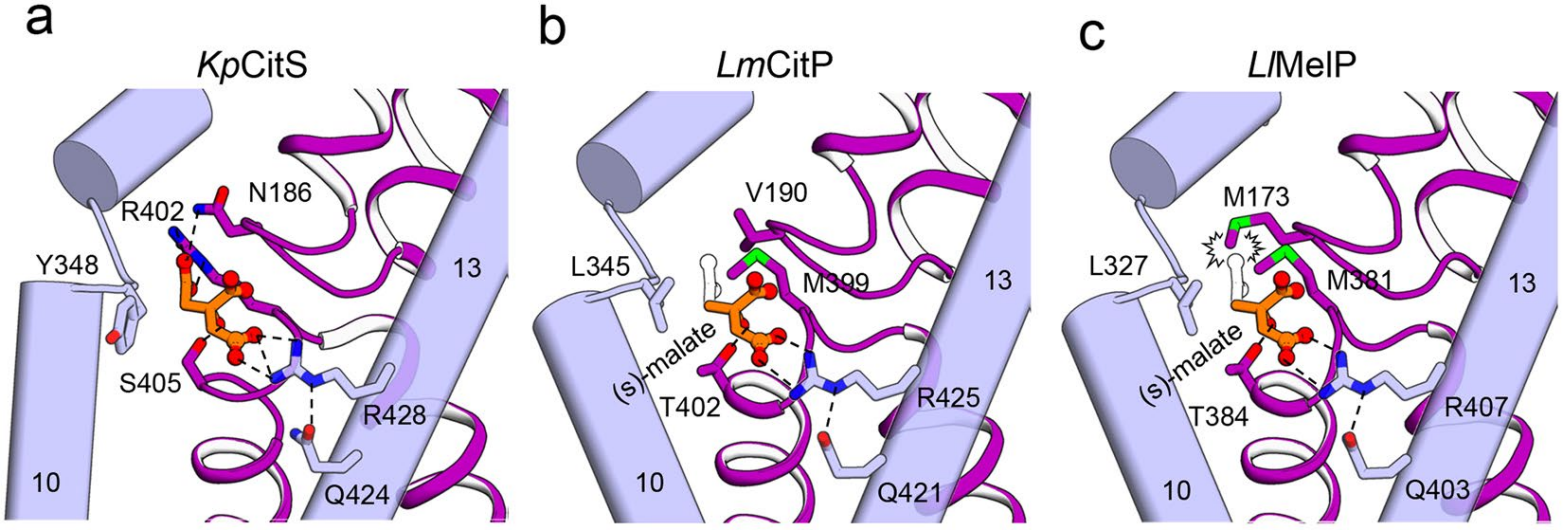
Homology models of *Lm*CitP and *Ll*MelP. The substrate binding sites of *Kp*CitS (**a**), and of homologous models of *Lm*CitP (**b**) and *Ll*MelP (**c**). Residues involved in substrate interaction are labeled. Hydrogen bonds and ionic interactions are shown by dashed lines. Backbone oxygens and nitrogens interacting with the substrate are not drawn. (S)-malates are shown as orange ball-and-stick models. The structures of the citrates superimposed on the malates are drawn as empty black sticks.

In order to understand the differences in substrate selectivity between *Kp*CitS and homologous 2-HCT proteins, we generated homology models of CitP of *Leuconostoc mesenteroides* (*Lm*CitP, citrate/lactate exchanger) and MleP of *Lactococcus lactis* (*Ll*MleP, malate/lactate exchanger)^1, 39^ based on the *Kp*CitS structures determined in this study. To increase the accuracy of the homology modeling, we excluded the N-terminus (1–46) and the linker region between TM 7 and TM 8 (237–265) of *Kp*CitS, in which there is low sequence conservation. The final models of *Lm*CitP and *Ll*MleP had similar folds to those of *Kp*CitS, with 31% and 33% sequence identity, and 56% and 43% similarity, respectively. In the modeled structures, S405 of *Kp*CitS is changed to threonine at positions 402 and 384 of *Lm*CitP and *Ll*MleP, respectively, where it mediates the invariant hydrogen bonding with the hydroxyl group of the 2-hydroxycarboxylate of citrate (Fig. 4). This is consistent with data, showing that removal of the hydroxyl group reduces the affinity for substrate of these proteins^1^. *Lm*CitP is known to transport both malate and citrate, but *Ll*MleP only transports malate^39^. Also, both transporters are highly stereoselective, that is, they favor substrates with (S)-enantiomeric rather than (R)-enantiomeric configurations. The modeled structures show that N186, Y348 and R402 in *Kp*CitS are replaced by V190, L345 and M399 in *Lm*CitP (Fig. 4, middle) and by M173, L327 and M381 in *Ll*MleP (Fig. 4, right). These alterations may affect the apparent affinity for substrate and substrate specificity by increasing the hydrophobicity and removing the anion-π interaction near the 3-carboxylate group of citrate, resulting in a shift of substrate preference towards (S)-malate. The modeled structure is consistent with previous biochemical data showing that mutation of N186 of *Kp*CitS to a valine severely reduces affinity for citrate with only minor changes in Vmax^40^. It is also consistent with functional data indicating that *Lm*CitP has ~40% higher activity for malate than for citrate^1^. In particular, M173 of *Ll*MleP is located in a position such that it can directly collide with the 3-carboxylate group of citrate, which accounts for why the latter is a substrate of *Lm*CitP but not of *Ll*MleP (Fig. 4, right)^39^.

## Discussion

We have studied the molecular mechanism of CitS in the 2-HCT family by determining multiple crystal structures along the transport cycle. Our results show that the *Kp*CitS has a homodimeric structure in which its dimerization domain remains fixed during the transport cycle due to a hydrogen bond network as well as extensive hydrophobic interactions, while the substrate-loaded transport domain undergoes an elevator-type translocation (i.e. up and down) across the membrane. Our crystal structures are fully consistent with previous functional evidence regarding the interaction between substrates and 2-HCT proteins. For example, site-directed mutagenesis demonstrated that conservative mutation of R428 to a positively charged lysine in *Lm*CitP and in CimH of *Bacillus subtilis* (*Bs*CimH, citrate/malate symporter), caused a 10-fold decrease in affinity for citrate. However, their replacement with uncharged cysteines had a more serious effect, reducing the affinity for citrate more than 20-fold^15, 41^. Reaction of the cysteine mutants with MTSEA (2-aminoethyl-methanethiosulfonate), which introduces a positive charge on the thiol groups of cysteine residues, resulted in 50-fold and 2-fold increases in transport activity, respectively^14, 15^. Conversely, the addition of a negative charge to the cysteine mutant of *Bs*CimH by reaction with MTSES (2-sulfonatoethyl-methanethiosulfonate) resulted in a 2-fold decrease in activity, highlighting the importance of electrostatic interactions between substrate and transporter. Our structural observations are also consistent with previous evidence that mutations of residues in HP1 and HP2 cause substantial reductions in the transport function of *Kp*CitS (Supplementary Fig. 10)^24, 25^. Locating these residues in our *Kp*CitS structure showed that they participate directly in either sodium binding or substrate coordination. Thus, mutation of these residues seems to result in reduced affinity for ions/substrate, or impede the elevator-like movement of the transport domain.

**Figure 5:**
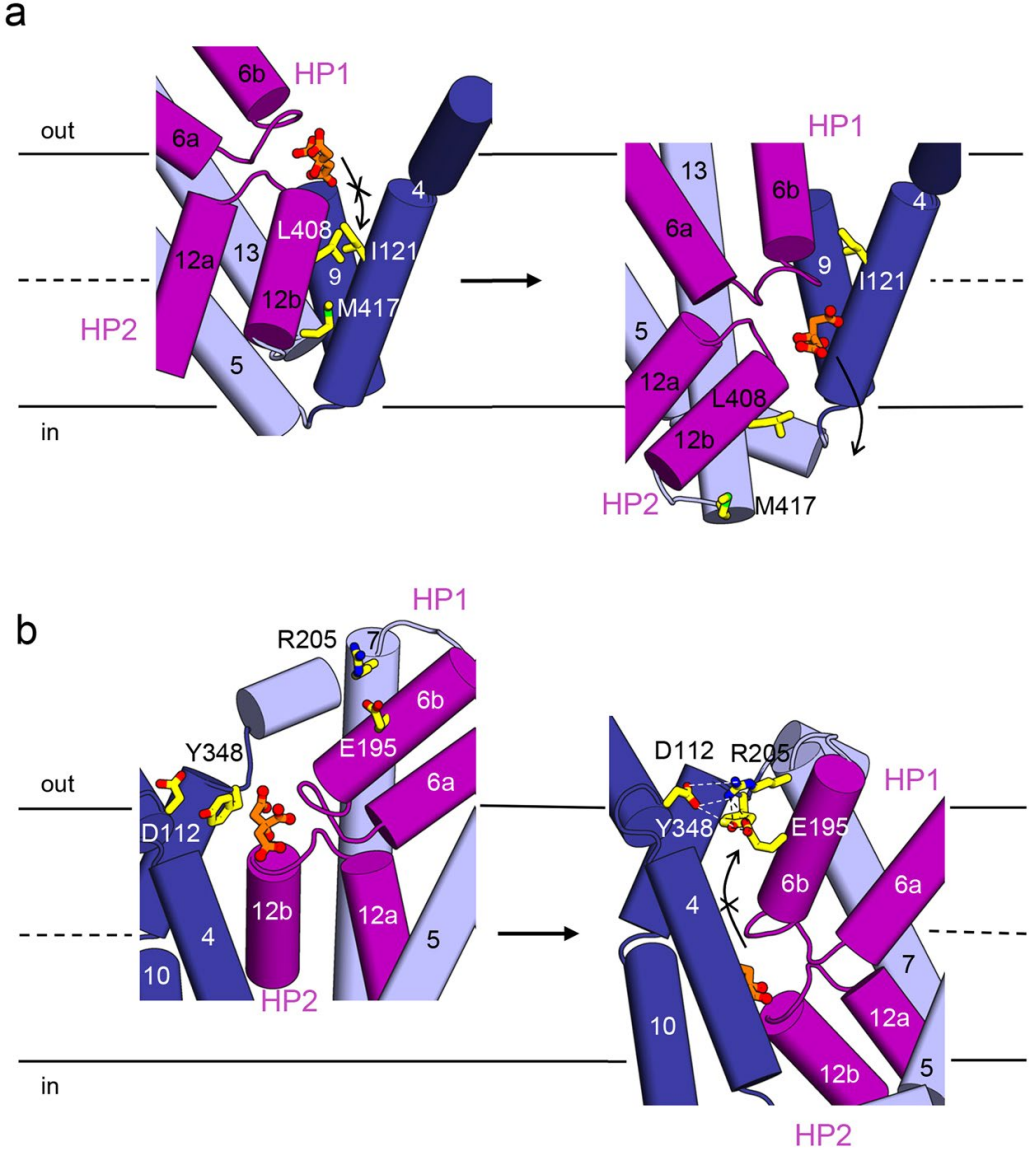
Alternating access mechanism of the *Kp*CitS. (**a**) In the outward-facing conformation, the transport pathway is blocked by I121, L408 and M417. In the inward-facing conformation, these residues move and citrate is released to the cytoplasm. The citrate is shown in orange and red. The release of citrate in the inward-facing conformation is marked by a black arrow. (**b**) Role of the polar residues in the alternating access mechanism. In the inward-facing conformation, D112, E195, R205 and Y348 form a salt bridge network at the membrane/periplasm interface, by virtue of which the substrate transport pathway is open only towards the cytoplasm. The view is rotated by 30 degrees along the vertical axis from that of (**a**).

From the *Kp*CitS structures, it is immediately evident how the internal substrate-binding site is alternatively accessible on one side and then the other of the membrane during the transport cycle (Fig. 1c)^42^. In the outward-facing conformation, L408 and I121 are in close proximity, and serve as a latch governing access of substrate to the cytoplasm (Fig. 5a). In addition, M417 seems to act as a plug preventing substrate exit. The conformational change to the inward-facing state relocates L408 and M417, opening the substrate binding site to the cytosol. Conversely, D112, E195, R205 and Y348 form a salt bridge network in the inward-facing conformation, blocking entry of substrate from the periplasm (Fig. 5b). A change of surface charge distribution also supports the alternating access mechanism of *Kp*CitS (Supplementary Fig. 11); the electrostatic surface potential at the periplasmic and cytoplasmic surfaces alternates between the positive and neutral charges along the transport cycle that will assist binding of the negatively charged citrate.

**Figure 6:**
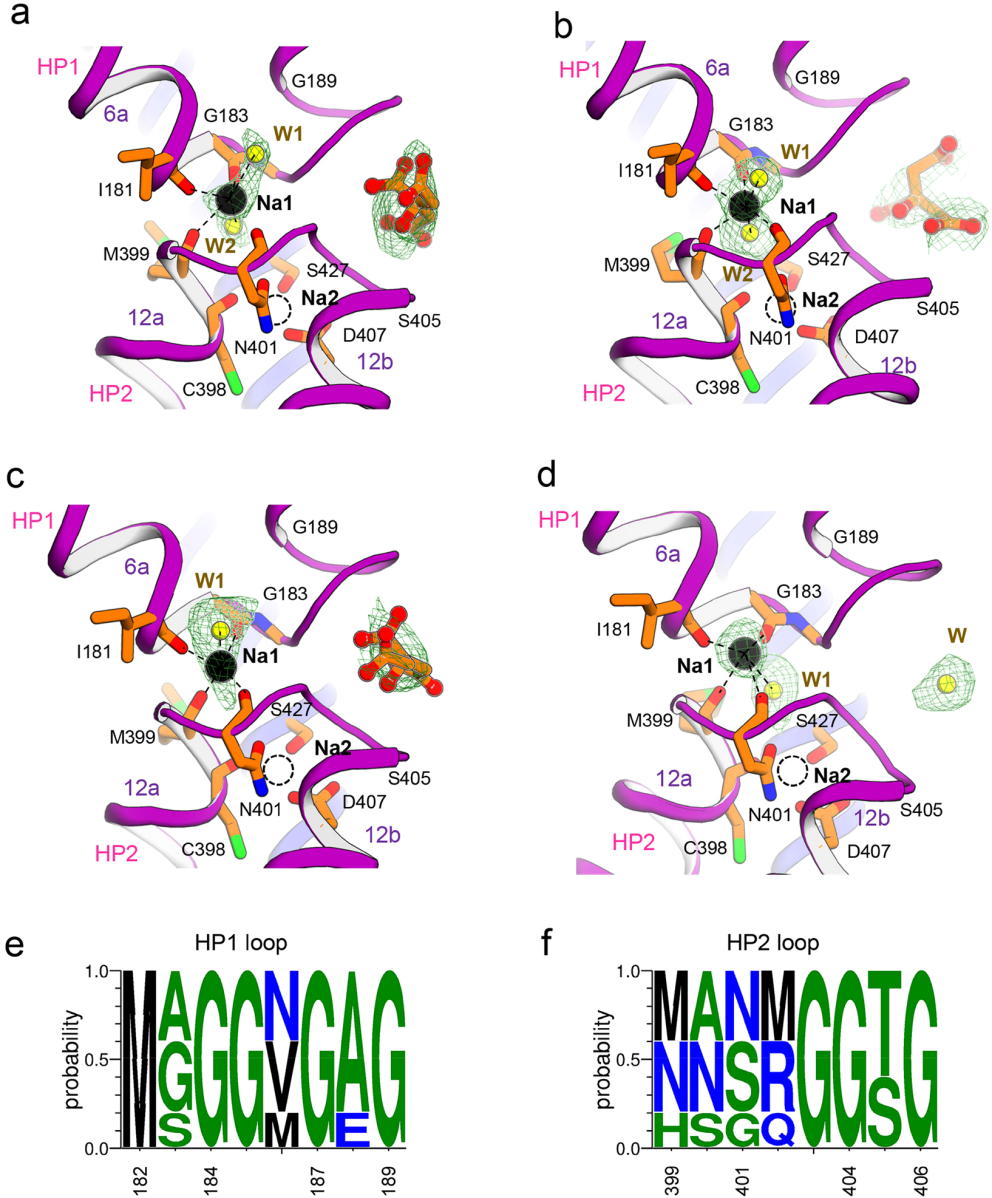
The sodium ion binding sites in *Kp*CitS. (**a**) The outward-facing protomer in the asymmetric conformation. (**b**) The inward-facing protomer in the asymmetric conformation. (**c**) The protomer in the outward-facing conformation. (**d**) The protomer in the inward-facing conformation. Citrate is shown as an orange ball-and-stick model, sodium ions (Na1) as black spheres, and water molecules (W, W1, W2) as yellow spheres. The empty Na2 sites are shown as dashed spheres. Hydrogen bonds and ionic interactions are drawn as dashed lines. The simulated annealing omit maps superimposed on the refined citrates, sodium ions, and water molecules were contoured at 2.8 σ level. (**e**,**f**) WebLogo representations of the sequence alignments and conservation in the HP1 and HP2 loops of 2-HCT proteins^54, 55^. The amino acid positions in the multiple sequence alignment are: *Kp*CitS (182–189, 399–406), *Se*CitS (182–189, 399–406), *Bs*CimH (191–198, 403–410), *Lm*CitP (186–193, 396–403), and *Ll*Melp (169–176, 378–385) for the HP1 and HP2 loops, respectively.

While the *Se*CitS structure revealed that two sodium ions (Na1 and Na2) are coupled to the substrate binding site^22^, we observed only Na1 between the two hairpin loops of *Kp*CitS in all three states (Fig. 6). Na1 is coordinated by the backbone oxygens of I181, G183, M399 and N401 with either an octahedral or a trigonal bipyramidal geometry with two and one ordered water molecule, respectively. As in the *Se*CitS structure, the proposed binding site for Na2 in *Kp*CitS is coordinated by side chains of S427, N401 and D407 and the backbone carbonyl oxygen of C398, but no electron density for Na2 was observed in either protomer (Fig. 6). There are three possible reasons why only one sodium ion is observed in each protomer of our structures. The first is that although Na2 is bound to the transporter, the resolution of the data is not high enough to identify its position. The second possibility is that Na1 is sufficient for binding of substrate, but not for transporting it in the outward-facing protomer. The crystal structure of *Kp*CitS shows five and three glycine residues, respectively, in the HP1 and HP2 loops (Fig. 6e,f). This is reminiscent of HP2 in GltPh^43^. As in that case, we imagine that the cluster of glycines is likely to confer high structural flexibility on the HP loops of *Kp*CitS, enabling its conformation to change easily to provide a perfect fit for a substrate molecule upon binding of one sodium ion. As *Kp*CitS has been shown to transport substrate coupled with two sodium ions^16, 19^, we think it is likely that the binding of Na1 precedes or is nearly simultaneous with that of substrate, and that Na2 is necessary only for substrate transport. The third possibility is that our *Kp*CitS structures may have been captured after Na2 had already been released from the inward-facing protomer into the cell. This idea is supported by many structures of secondary active transporters as well as their MD simulations, which show that substrate release is initiated by dissociation of a sodium ion^4, 16, 22, 31, 32, 44–48^.

**Figure 7:**
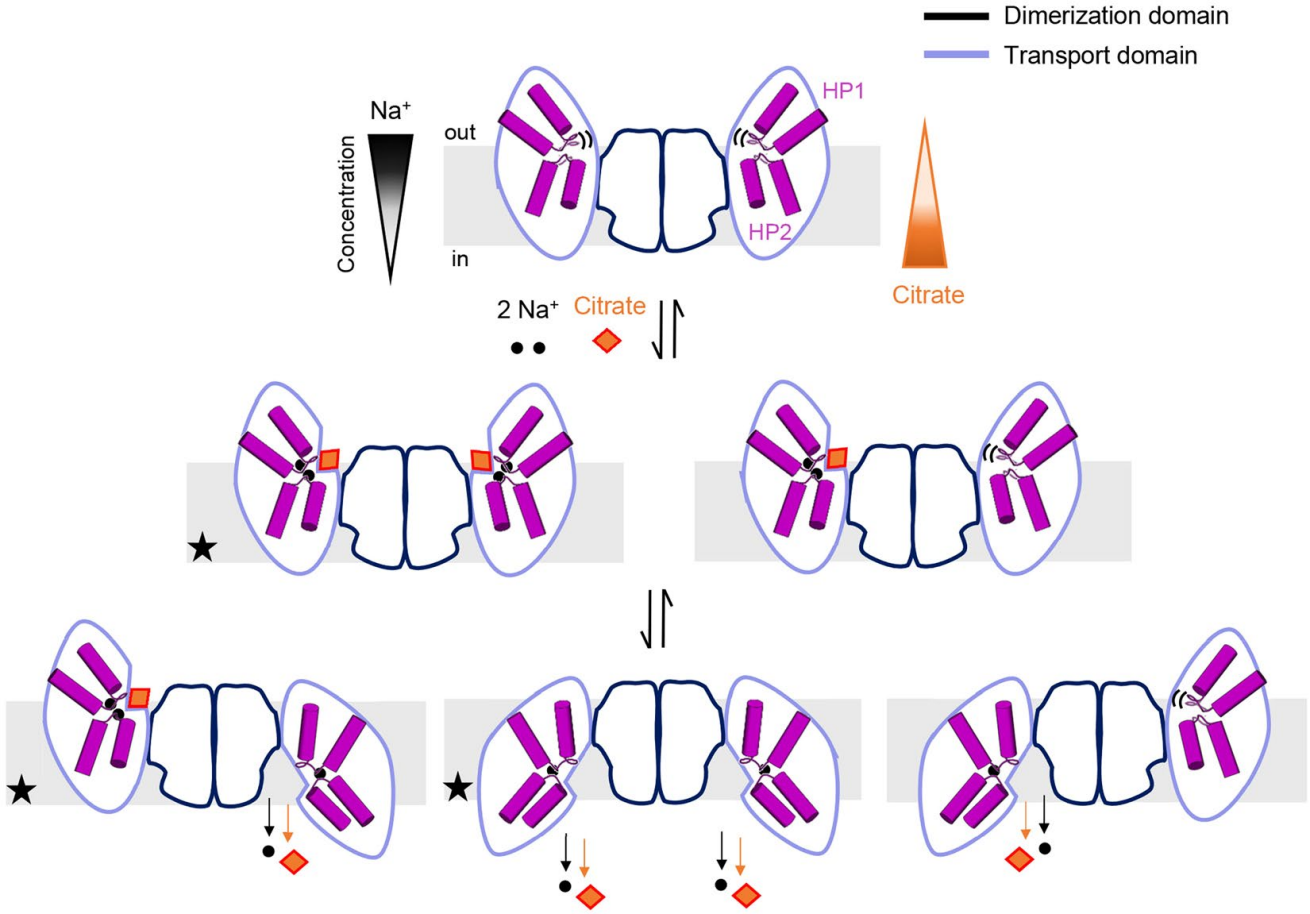
Proposed transport mechanism of the CitS. Dimerization and transport domains are shown in dark and pale blue, respectively. The helical hairpins, HP1 and HP2, of the transport domain are represented as cylinders and loops in purple. Citrate is shown as an orange diamond, and sodium ions as black spheres. In the apo state, the binding site of substrate and ions is open toward the external environment. Binding of sodium ions prepares the protein to interact with its substrate, which promotes the elevator-like movement of the transport domain in either one of two protomers in the dimer or both. Dissociation of citrate and ions into the cytoplasm resets the protein into the outward-facing apo state. The crystal structures of CitS that have been determined by us and others^22^ are marked with black stars.

The structural data for *Kp*CitS, combined with the previous results of diverse experimental approaches, allow us to propose a molecular mechanism for the CitS in the 2-HCT family^4, 16, 19, 24, 42^ (Fig. 7). In this model, the outward-facing conformation is in the apo state with substrate and sodium ions unbound. When the protein is occupied by sodium ions, it presents a suitable binding site for substrate, and undergoes a rotational and vertical movement across the membrane. This elevator-like movement is accompanied by changes of amino acid conformation essential for substrate binding and hydration of the substrate binding site by water influx, resulting in disruption of the protein-substrate interaction, release of substrate and sodium ions, and return to the apo state. Unfortunately, current structural data do not clearly address whether this process occurs in both of the protomers at the same time, whether it occurs in only one protomer, or whether it occurs alternately in the two protomers^16^ (Fig. 7).

In summary, we report the first crystal structures of *Kp*CitS in different functional states. Our crystal structures, together with the previously reported structural data, provide evidence that the elevator-type alternating access mechanism is widespread among secondary active transporters^22, 34, 38, 49–51^. In essence we propose that CitS proteins employ a sodium dependent elevator-like movement in which the dimerization domain is held fixed during the transport cycle, and the transport domain moves up and down across the membrane bilayer to translocate the substrate. Further research is required to unravel many details of this model, such as how substrate transport by 2-HCT proteins is coupled to the ion gradient and/or ion binding^52^.

## Electronic supplementary material

Dataset1
