# Supplementary material for: Structural insights into the elevator-like mechanism of the sodium/citrate symporter CitS
Source: Sci Rep. 2017 May 31;7:2548. doi: 10.1038/s41598-017-02794-x (PMC5451387; doi:10.1038/s41598-017-02794-x)
Supplement: Supplementary file 1 — Dataset1 [file 41598_2017_2794_MOESM1_ESM.doc]

**Supplementary Information**

**Structural insights into the elevator-like mechanism of the sodium/citrate symporter CitS**

Ji Won Kim^1^*, Subin Kim^2^*, Songwon Kim^2^, Haerim Lee^1^, Jie-Oh Lee^1^ & Mi Sun Jin^2^

^1^Department of Chemistry, KAIST, 291 Daehak-ro, Yuseong-gu, Daejeon 34141, Republic of Korea; ^2^School of Life Sciences, GIST, 123 Cheomdan-gwagiro, Buk-gu, Gwangju 61005, Republic of Korea

**Supplementary Discussion**

**Cross-linking analysis**

To confirm the proposed role of the hinges in stabilizing the *Kp*CitS dimer, we performed a glutaraldehyde cross-linking experiment in which five residues in *Kp*CitS (D50, S114, N115, R266, Q335) were changed to alanines, individually and in combination, by site-direct mutagenesis (Supplementary Fig. 8a, b). Most of the single, double and triple mutants had similar levels of expression to wild-type *Kp*CitS (Supplementary Fig. 8c, d), and treatment with glutaraldehyde caused complete disappearance of the 37 kDa band corresponding to the *Kp*CitS monomer on SDS-PAGE, and the appearance of a single strong band corresponding to the dimer (approximately 80 kDa). In contrast, exposure of SDS-treated *Kp*CitS to glutaraldehyde resulted in no shift to the dimer mass on SDS-PAGE, demonstrating that the cross-linking by glutaraldehyde depended on the presence of a stable dimeric complex rather than being due to artifactual aggregation. However, the expression of proteins with quadruple and quintuple mutations, with one exception, was very low or completely absent, and glutaraldehyde barely altered their migration (Supplementary Fig. 8e). Thus, together, the structural and cross-linking data suggest that in addition to the hydrophobic interactions, residues in the hinges contribute in part to stabilize the dimers, and that interfering with this stabilization abolishes transport function either due to exposure of the hydrophobic interface to solvent or to formation of poorly packed dimers.

**Methods**

**Design of the DNA construct, expression of the protein and purification**

The *Kp*CitS protein was prepared as previously described[^40^](#_ENREF_40). Briefly, a synthetic gene encoding *Kp*CitS (Bioneer Inc.) was cloned between the NdeI and BamHI restriction enzyme sites in a modified pET16b vector incorporating an N-terminal 10XHis tag followed by a thrombin protease site. Originaly we constructed the full-length *Kp*CitS, but we observed that the first N-terminal residues of *Kp*CitS were cleaved heterogeneously by thrombin protease action. To increase the stability and homogeneity of the protein, we replaced the ^7^PPATEK^12^ amino acid region of *Kp*CitS with a thrombin recognition sequence (LVPRGS).

The protein was produced in *Escherichia coli* C43(DE3) cells. The cells were cultured in Terrific Broth at 30 °C; when the absorbance at 600 nm was 0.6 they were induced with 0.7 mM isopropyl-β-d-thiogalactopyranoside (IPTG) for 20 hours at 25 °C. After harvesting by centrifugation at 9,000 g for 15 minutes, the cells were frozen in liquid nitrogen and broken by a microfluidizer in a lysis buffer containing 20 mM Tris-HCl pH 7.5, 300 mM NaCl, 100 mM DNaseI and 0.1 mM phenylmethylsulfonyl fluoride (PMSF). Cell debris was removed by centrifugation at 2,000 g for 15 minutes, and the supernatant was centrifuged at 90,000 g for 1 hour to collect membranes containing *Kp*CitS. The membranes were solubilized with 2% (w/v) n-dodecyl-β-D-maltopyranoside (DDM; Affimetrix) or with 3% (w/v) n-decyl-β-D-maltopyranoside (DM; Affimetrix) in the lysis buffer with 5 mM MgCl_2_ for 2 hours at 4 °C. Unsolubilized membranes were removed by centrifugation at 90,000 g for 30 minutes, and the supernatant was loaded onto nickel affinity resin (Incospharm, Korea) equilibrated with 20 mM Tris-HCl pH 7.5, 300 mM NaCl, 70 mM imidazole and 0.15% (w/v) DM. Protein was eluted with 20 mM Tris-HCl pH 7.5, 300 mM NaCl, 500 mM imidazole and 0.15% (w/v) DM, and excess imidazole was immediately removed with a PD-10 desalting column (GE Healthcare). *Kp*CitS was concentrated to 5 mg/ml with an Amicon ultracentrifugal filter (Millipore, MWCO 50K) and cleaved with 0.1% (w/w) thrombin protease overnight at 4 °C. The protein was further purified by Superdex 200 size exclusion chromatography (GE Healthcare) in a buffer containing 20 mM Tris-HCl pH 7.5, 150 mM NaCl, and 0.15% DM. Fractions containing *Kp*CitS were pooled and concentrated to 8 mg/ml.

**Site-directed mutagenesis and glutaraldehyde cross-linking**

Site-directed mutagenesis was performed by overlap polymerase chain reaction (PCR) to test the proposed role of the hinges in stabilizing *Kp*CitS dimers. All the mutations were confirmed by sequence analysis. For glutaraldehyde-mediated cross-linking, purified wild-type and mutant proteins were diluted to a concentration of 0.4 mM in a buffer containing 20 mM HEPES pH 7.5, 300 mM NaCl and 0.1% DDM, and mixed with 0.25 M glutaraldehyde solution (Sigma-Aldrich). The mixtures were incubated for 30 minutes at 4 °C, and reactions were quenched by addition of 20 mM Tris pH 8.0. The treated proteins were then analyzed by SDS-PAGE. As a negative control, 0.1% SDS was added to wild-type *Kp*CitS, and the mixture was incubated for 5 minutes at 4 °C before addition of glutaraldehyde (Lanes 3 and 4 in [Supplementary](../../../../C:%5Csupplementry) Fig. 8c).

**Crystallization and data collection**

The concentrated *Kp*CitS was supplemented with 1% (w/v) n-octyl-β-D-glucopyranoside (OG; Affimetrix) and incubated on ice for 30 minutes. In addition, 5 mM sodium citrate pH 7.5 was added for the outward-facing and the asymmetric conformations. Crystals of *Kp*CitS were grown at 4 °C by the sitting-drop vapor diffusion method after mixing 0.6 μl of protein solution with 0.3 μl of reservoir solution containing 100 mM MES pH 6.0-6.5, 100-200 mM NaCl and 32-36% PEG400. Crystals were dehydrated by 5% increment of PEG concentration to a final concentration of 37-40% in the reservoir over 12 hours. The crystals were flash-frozen in liquid nitrogen with Al´s oil as a cryoprotectant. Diffraction datasets were collected at the 5C and 7A beamlines of the Pohang Accelerator Laboratory (PAL) and at the 23-ID beamline of the Advanced Photon Source (APS). The datasets were indexed, integrated and scaled with the HKL2000 program package (HKL Research Inc.).

**Structure Determination and homology modeling**

The three *Kp*CitS structures were determined by molecular replacement (Phaser, CCP4)[^56^](#_ENREF_56) using the separate protomers of the *Se*CitS structure (PDB code 5A1S) as search probes[^22^](#_ENREF_22). The atomic models were constructed by iterative building in COOT[^57^](#_ENREF_57), and refined by CNS[^58^](#_ENREF_58), REFMAC5 with TLS[^59^](#_ENREF_59), and PHENIX program suits[^60^](#_ENREF_60). The X-ray crystallographic data and refinement statistics are summarized in Supplementary Table 1 and 2. The residues at the N-terminus and in the loop regions connecting TM 7 and TM 8 were disordered in the crystals, and were not included in the final structures. The homology models of *Lm*CitP and *Ll*MleP were constructed by the program MODELLER based on the structure of *Kp*CitS (http://salilab.org/modeller/about_modeller.html)[^61^](#_ENREF_61). All figures in the manuscript were generated using the program PyMOL (www.pymol.org).

**
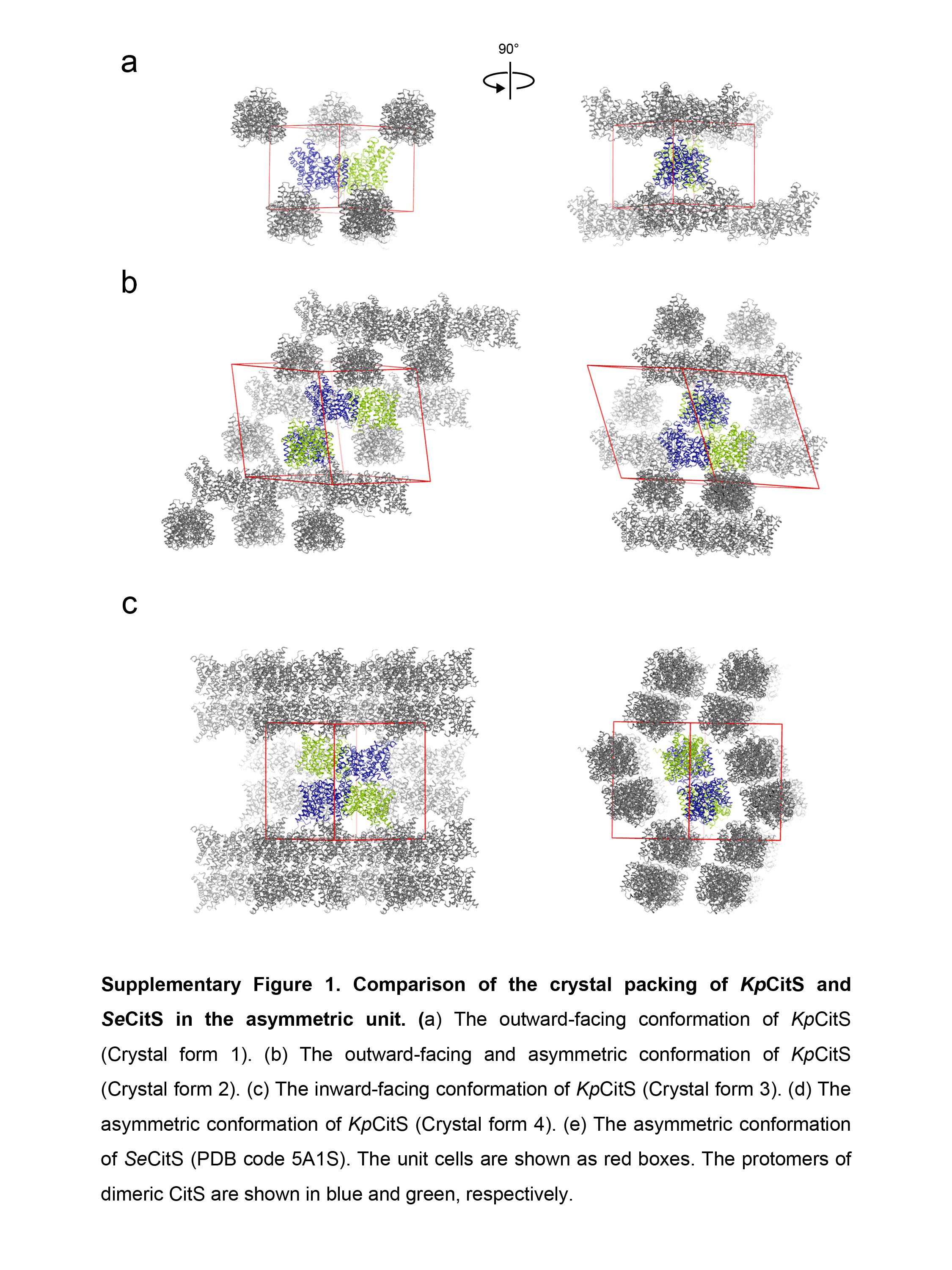
**

**Supplementary Figure 1. Comparison of the crystal packing of *Kp*CitS and *Se*CitS in the asymmetric unit. (**a) The outward-facing conformation of *Kp*CitS (Crystal form 1). (b) The outward-facing and asymmetric conformation of *Kp*CitS (Crystal form 2). (c) The inward-facing conformation of *Kp*CitS (Crystal form 3). (d) The asymmetric conformation of *Kp*CitS (Crystal form 4). (e) The asymmetric conformation of *Se*CitS (PDB code 5A1S). The unit cells are shown as red boxes. The protomers of dimeric CitS are shown in blue and green, respectively.

**Supplementary Figure 1 (Continued)**

**
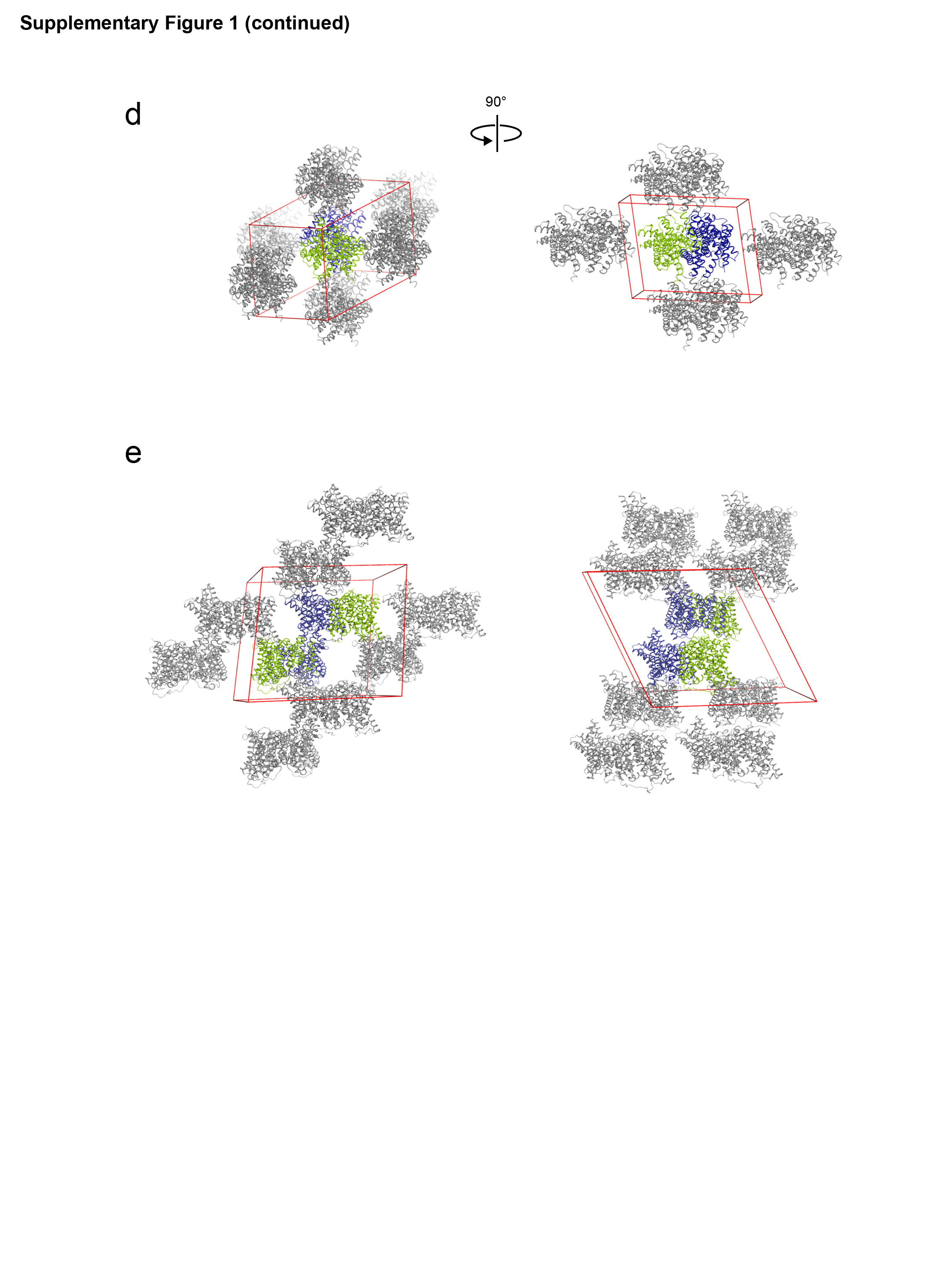
**

**
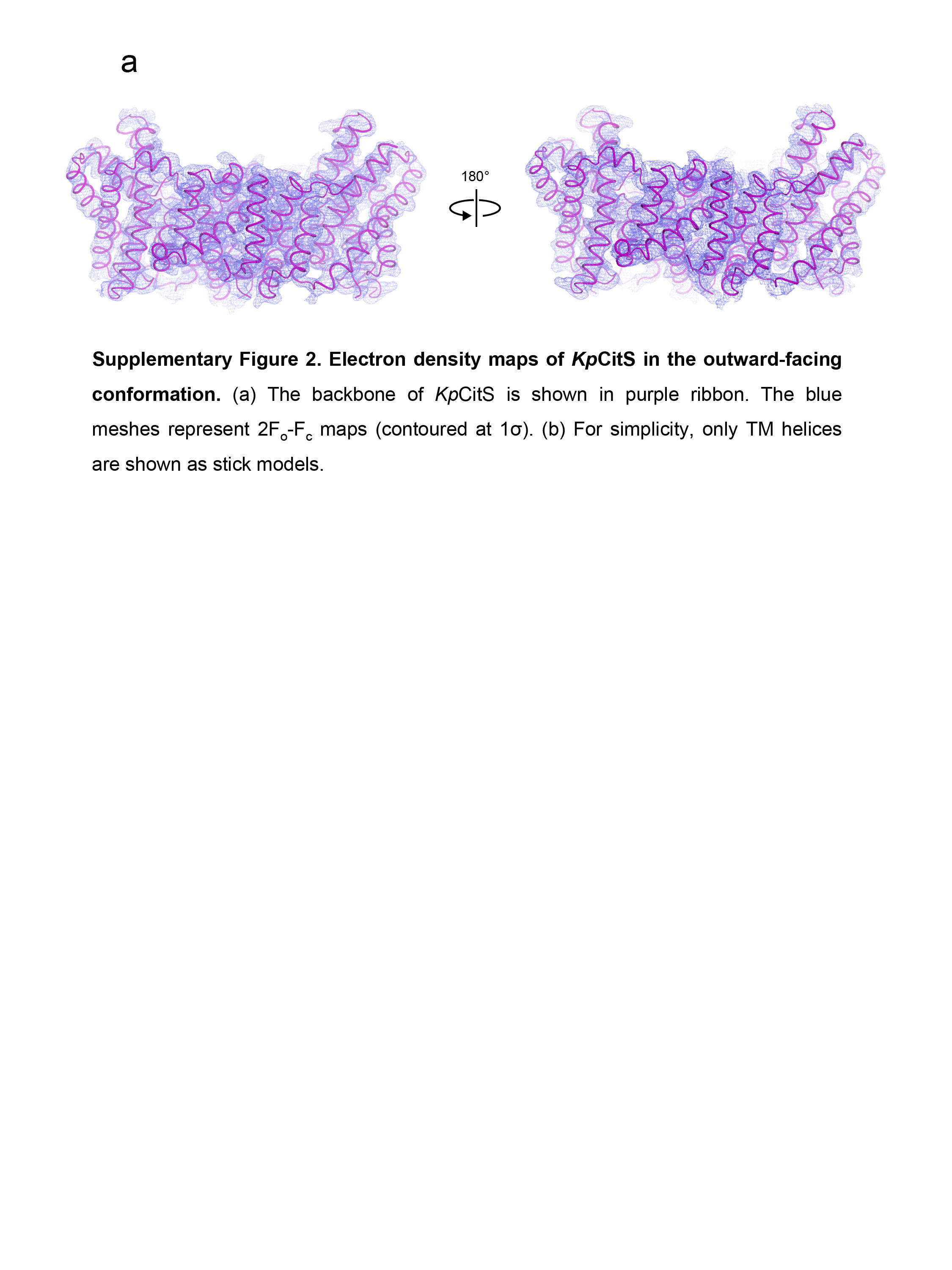
**

**Supplementary Figure 2. Electron density maps of *Kp*CitS in the outward-facing conformation.** (a) The backbone of *Kp*CitS is shown in purple ribbon. The blue meshes represent 2F_o_-F_c_ maps (contoured at 1σ). (b) For simplicity, only TM helices are shown as stick models.

**Supplementary Figure 2 (Continued)**

**
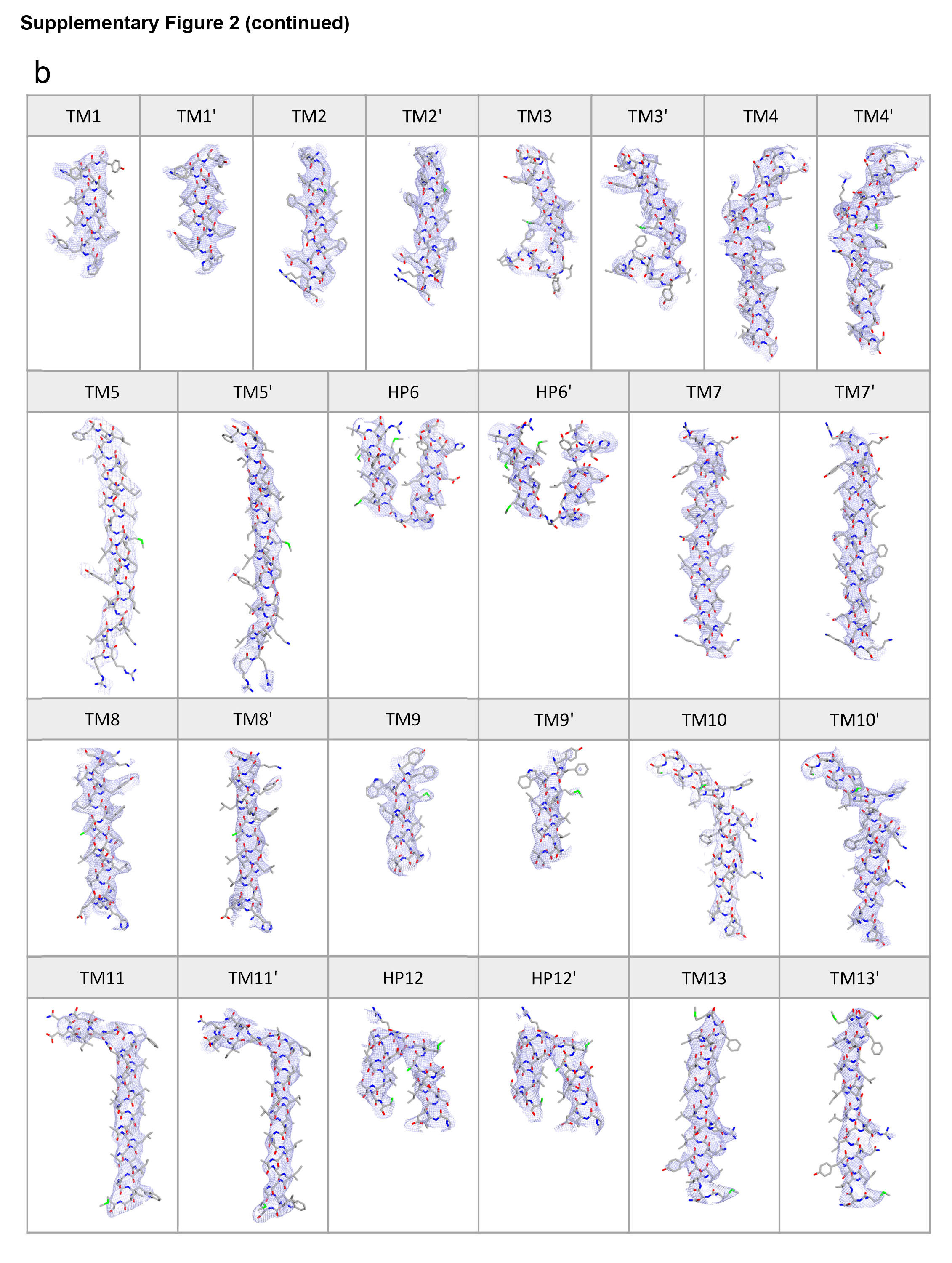
**

**
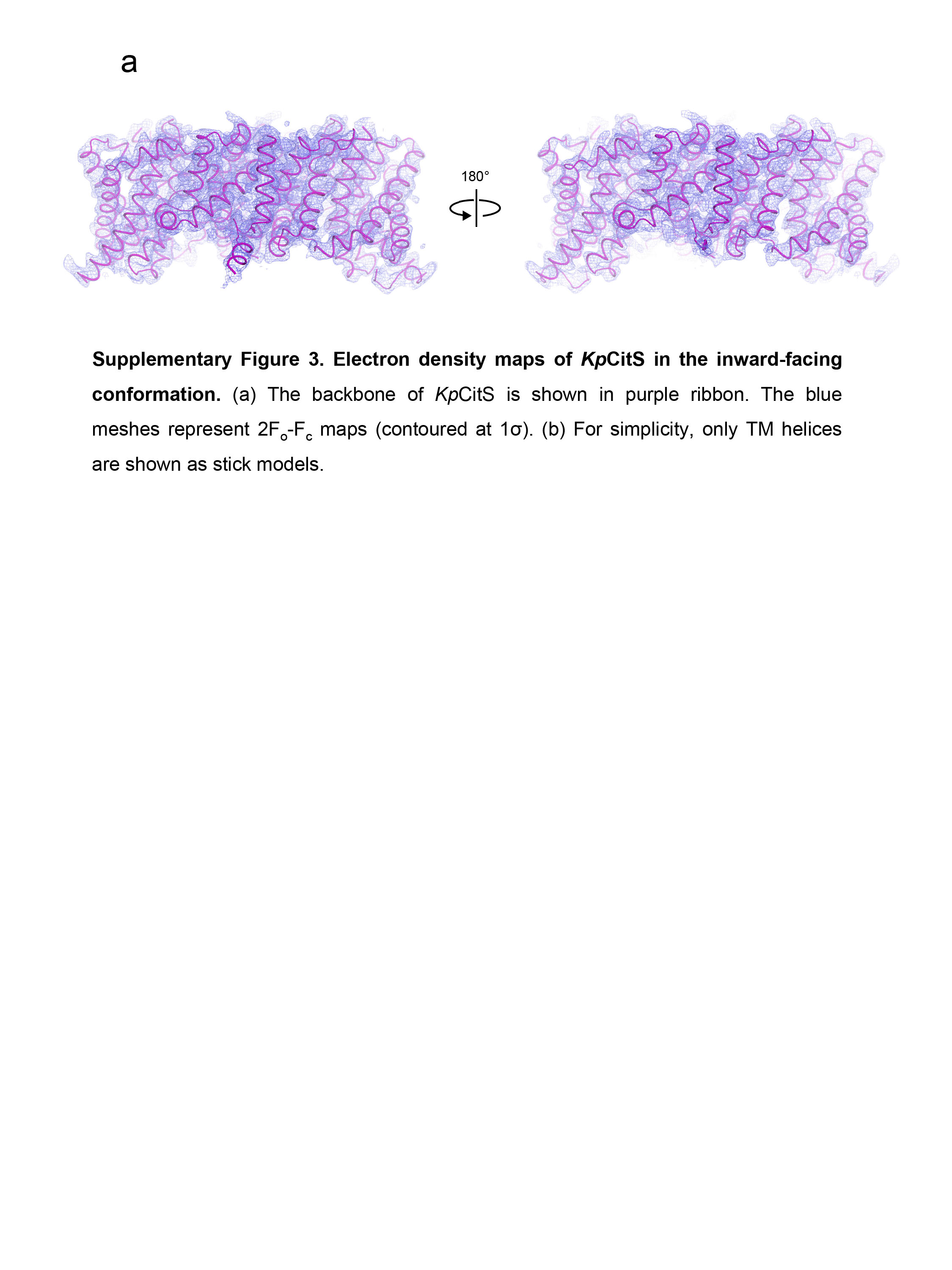
**

**Supplementary Figure 3. Electron density maps of *Kp*CitS in the inward-facing conformation.** (a) The backbone of *Kp*CitS is shown in purple ribbon. The blue meshes represent 2F_o_-F_c_ maps (contoured at 1σ). (b) For simplicity, only TM helices are shown as stick models.

**Supplementary Figure 3 (Continued)**

**
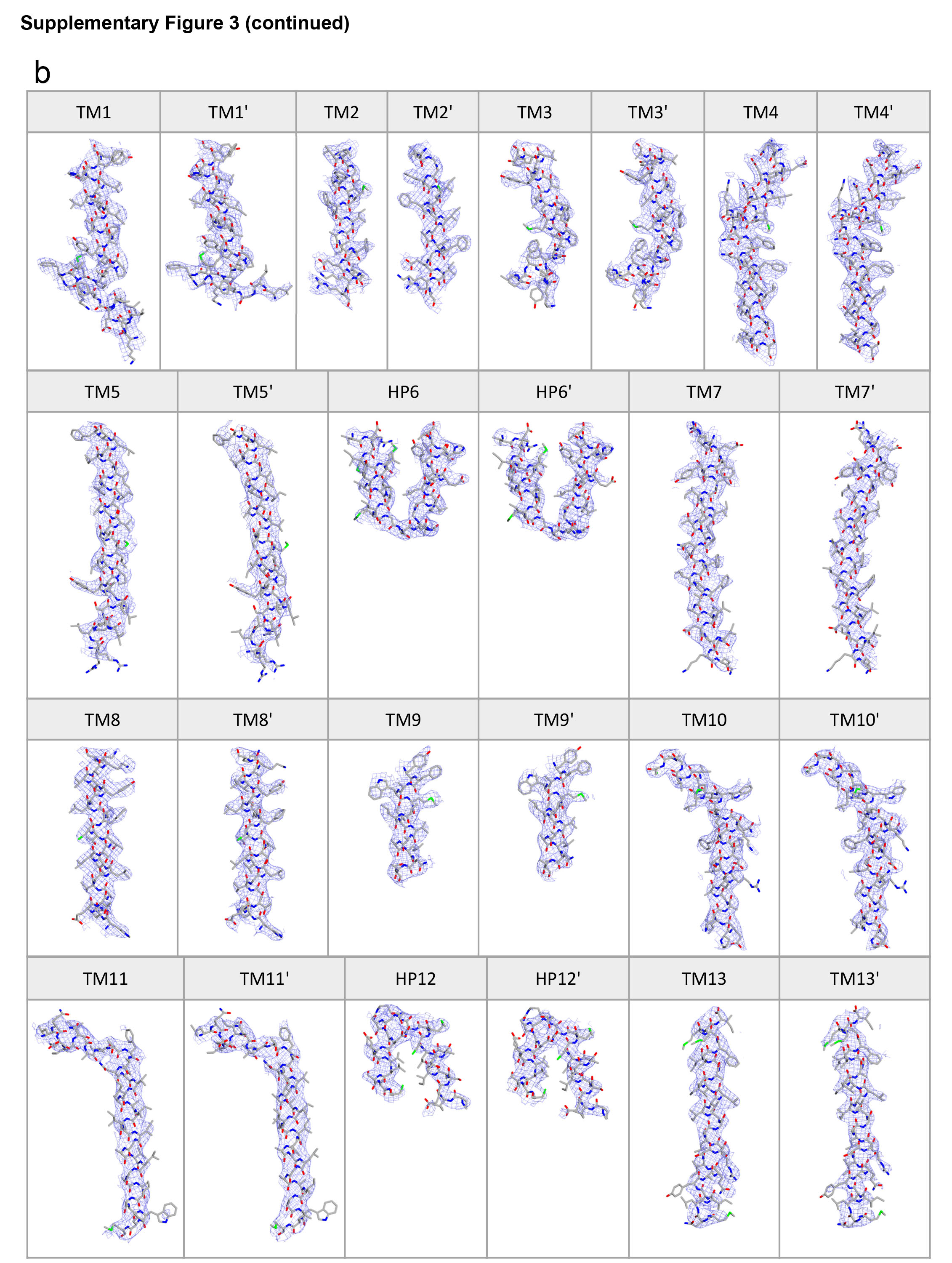
**

**
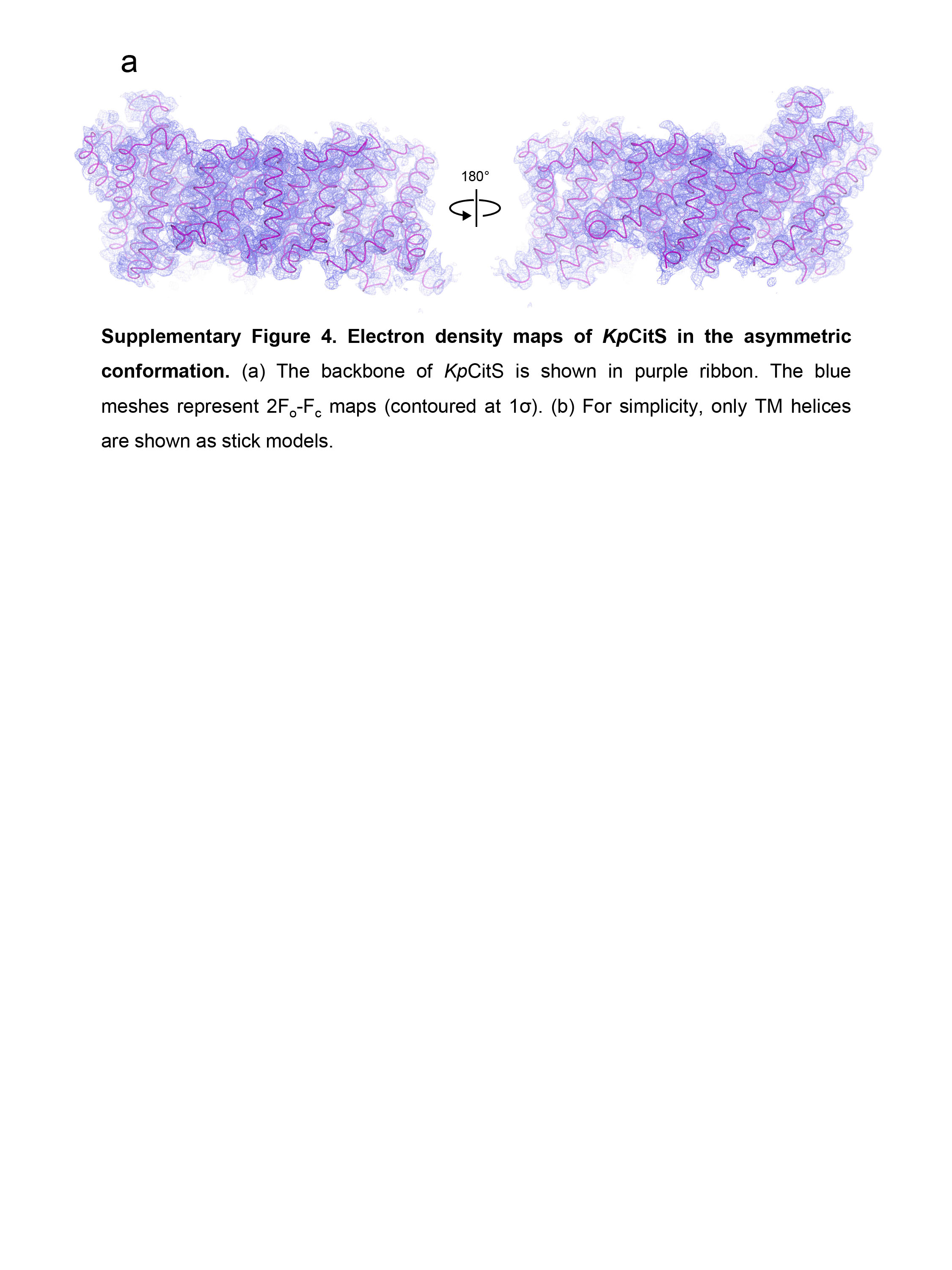
**

**Supplementary Figure 4. Electron density maps of *Kp*CitS in the asymmetric conformation.** (a) The backbone of *Kp*CitS is shown in purple ribbon. The blue meshes represent 2F_o_-F_c_ maps (contoured at 1σ). (b) For simplicity, only TM helices are shown as stick models.

**Supplementary Figure 4 (Continued)**

**
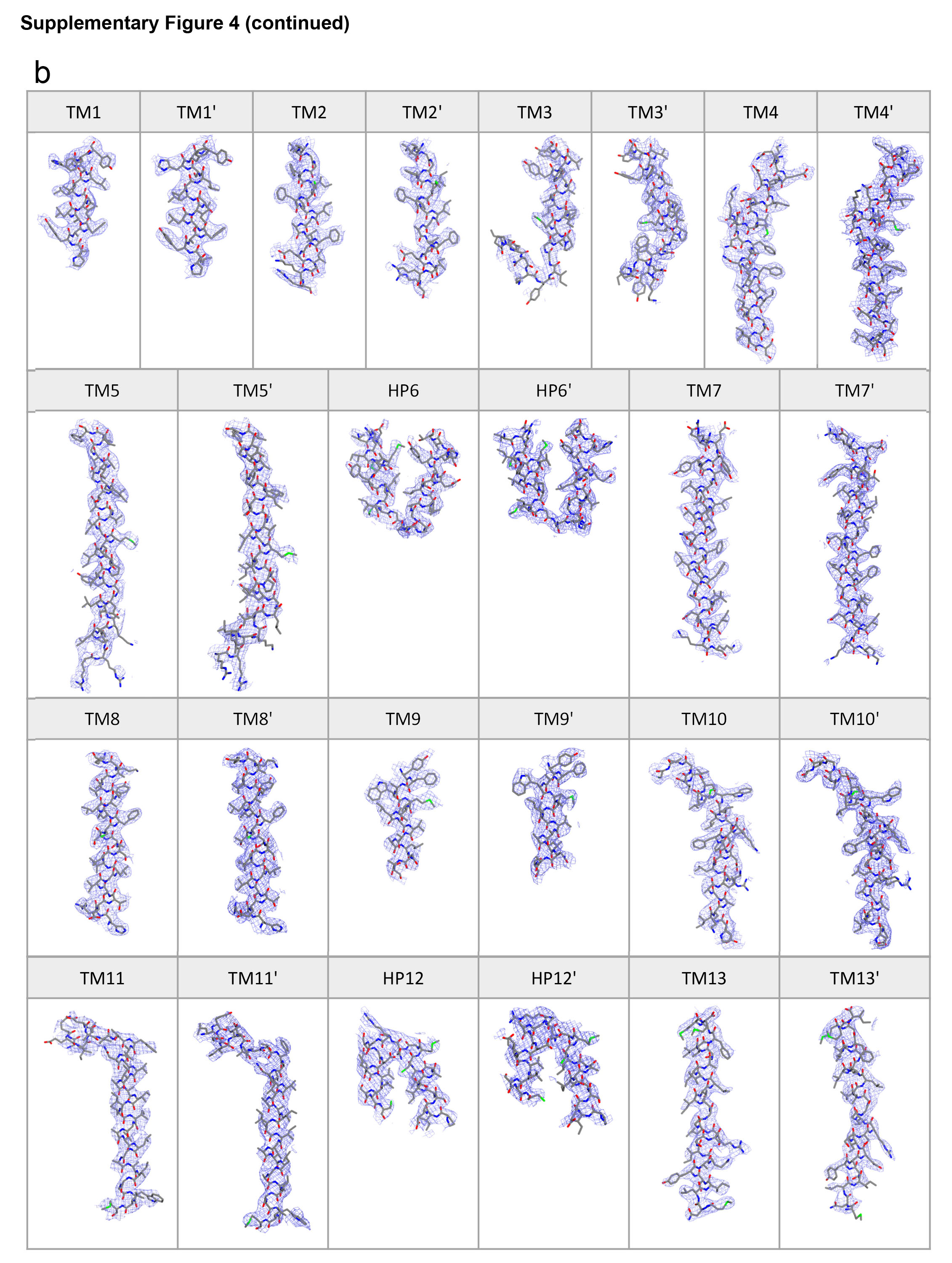
**

**
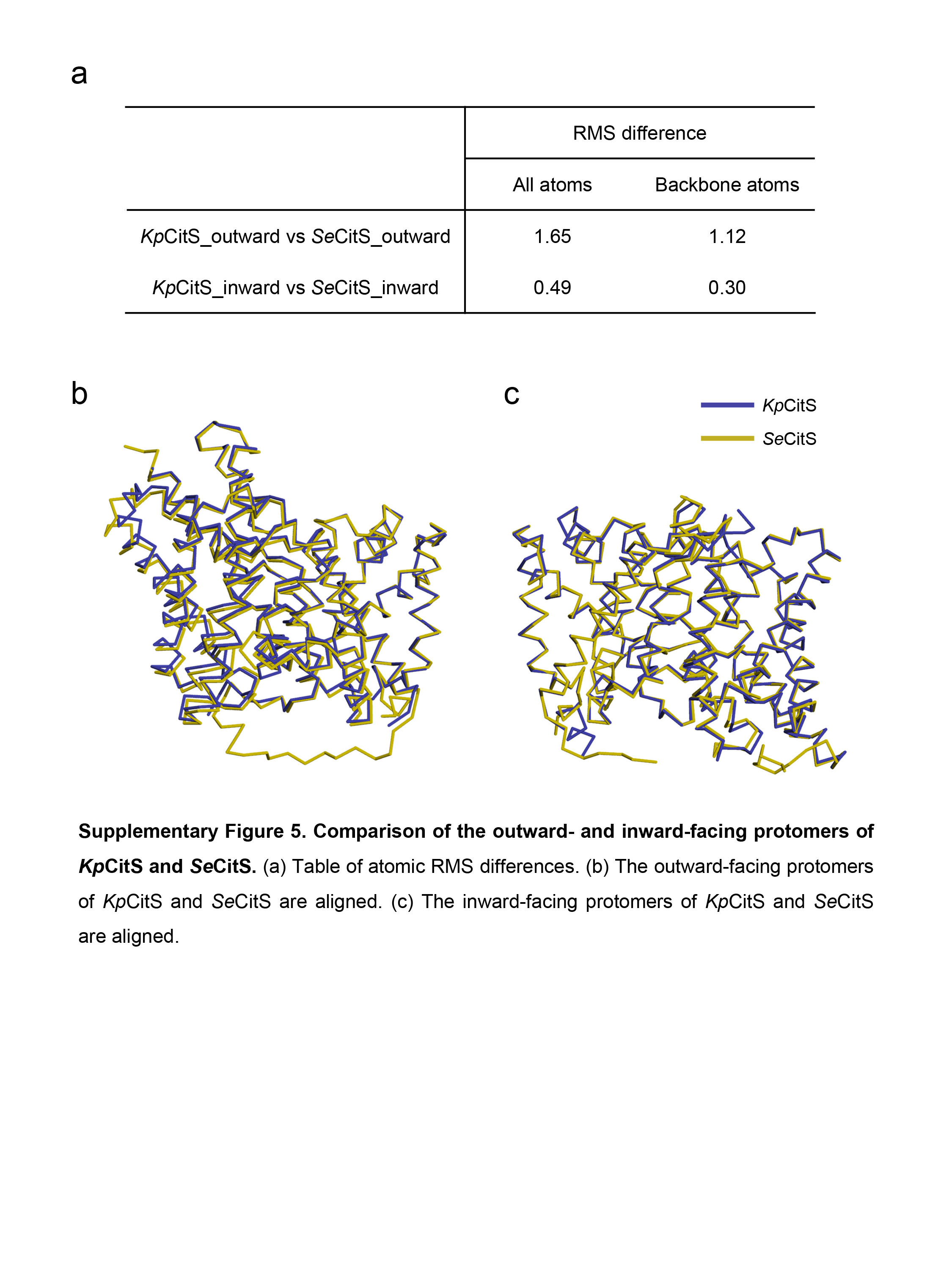
**

**Supplementary Figure 5. Comparison of the outward- and inward-facing protomers of *Kp*CitS and *Se*CitS.** (a) Table of atomic RMS differences. (b) The outward-facing protomers of *Kp*CitS and *Se*CitS are aligned. (c) The inward-facing protomers of *Kp*CitS and *Se*CitS are aligned.

**
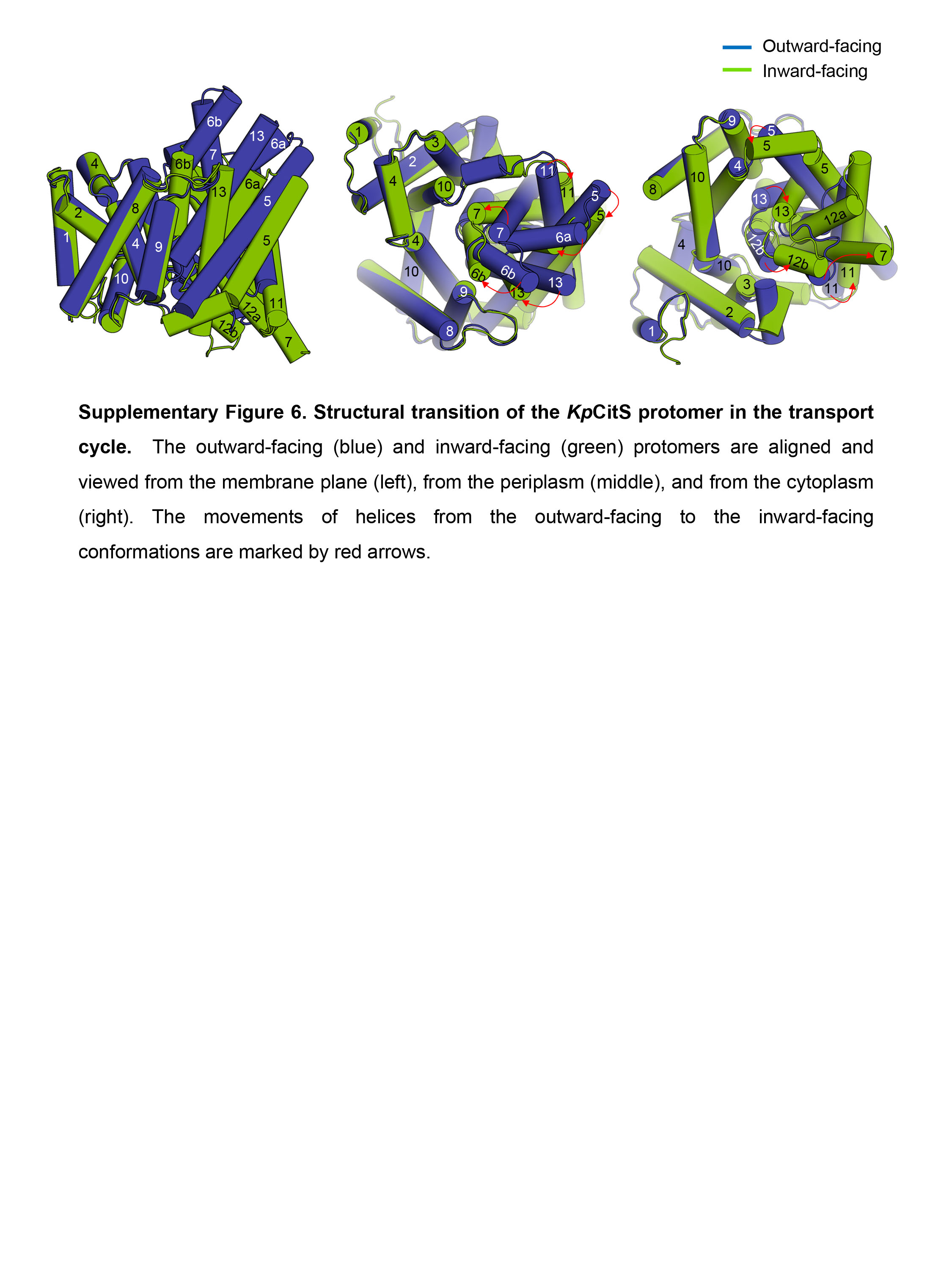
**

**Supplementary Figure 6. Structural transition of the *Kp*CitS protomer in the transport cycle.**  The outward-facing (blue) and inward-facing (green) protomers are aligned and viewed from the membrane plane (left), from the periplasm (middle), and from the cytoplasm (right). The movements of helices from the outward-facing to the inward-facing conformations are marked by red arrows.

**
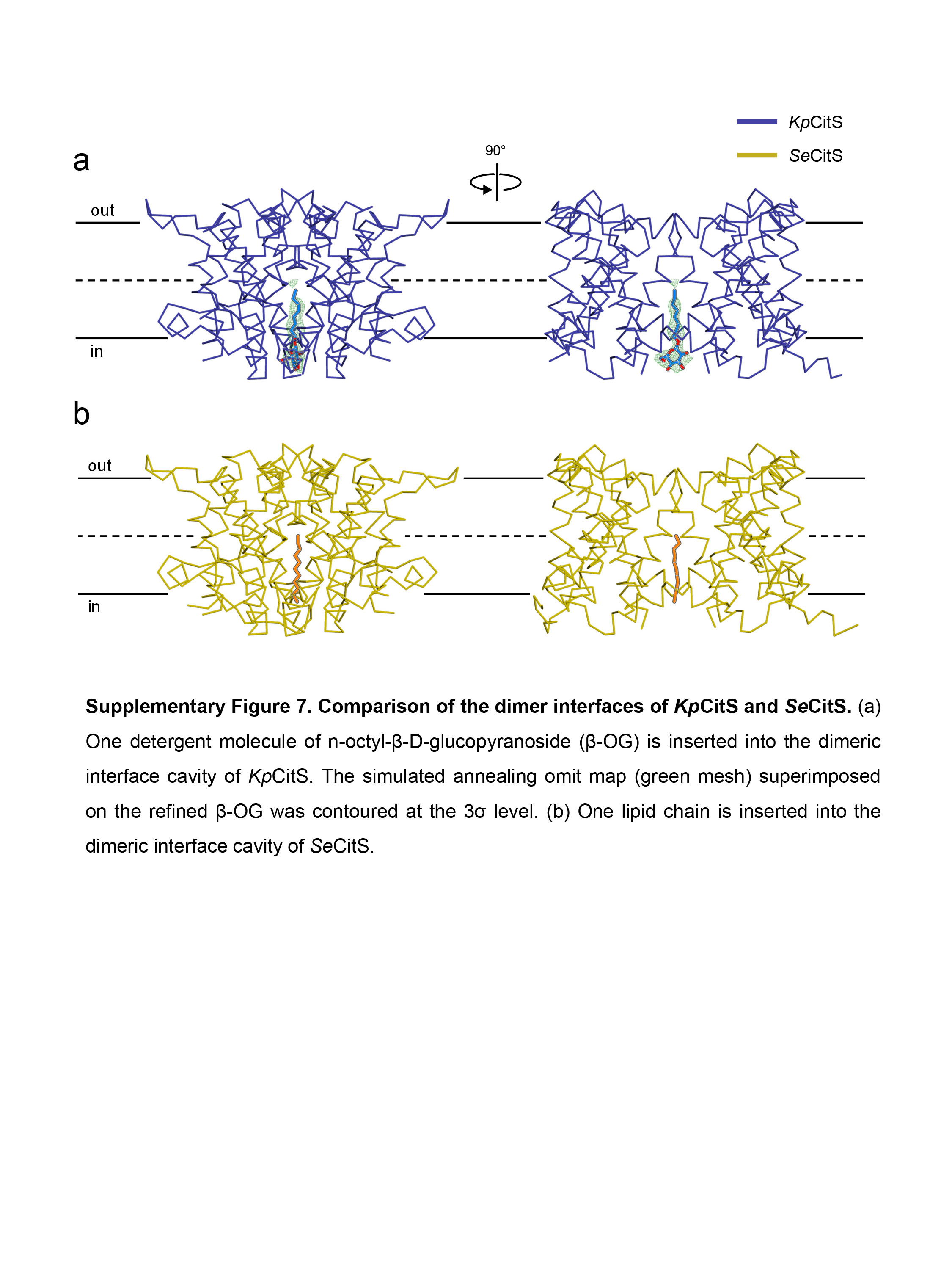
**

**Supplementary Figure 7. Comparison of the dimer interfaces of *Kp*CitS and *Se*CitS.** (a) One detergent molecule of n-octyl-β-D-glucopyranoside (β-OG) is inserted into the dimeric interface cavity of *Kp*CitS. The simulated annealing omit map (green mesh) superimposed on the refined β-OG was contoured at the 3σ level. (b) One lipid chain is inserted into the dimeric interface cavity of *Se*CitS.

**
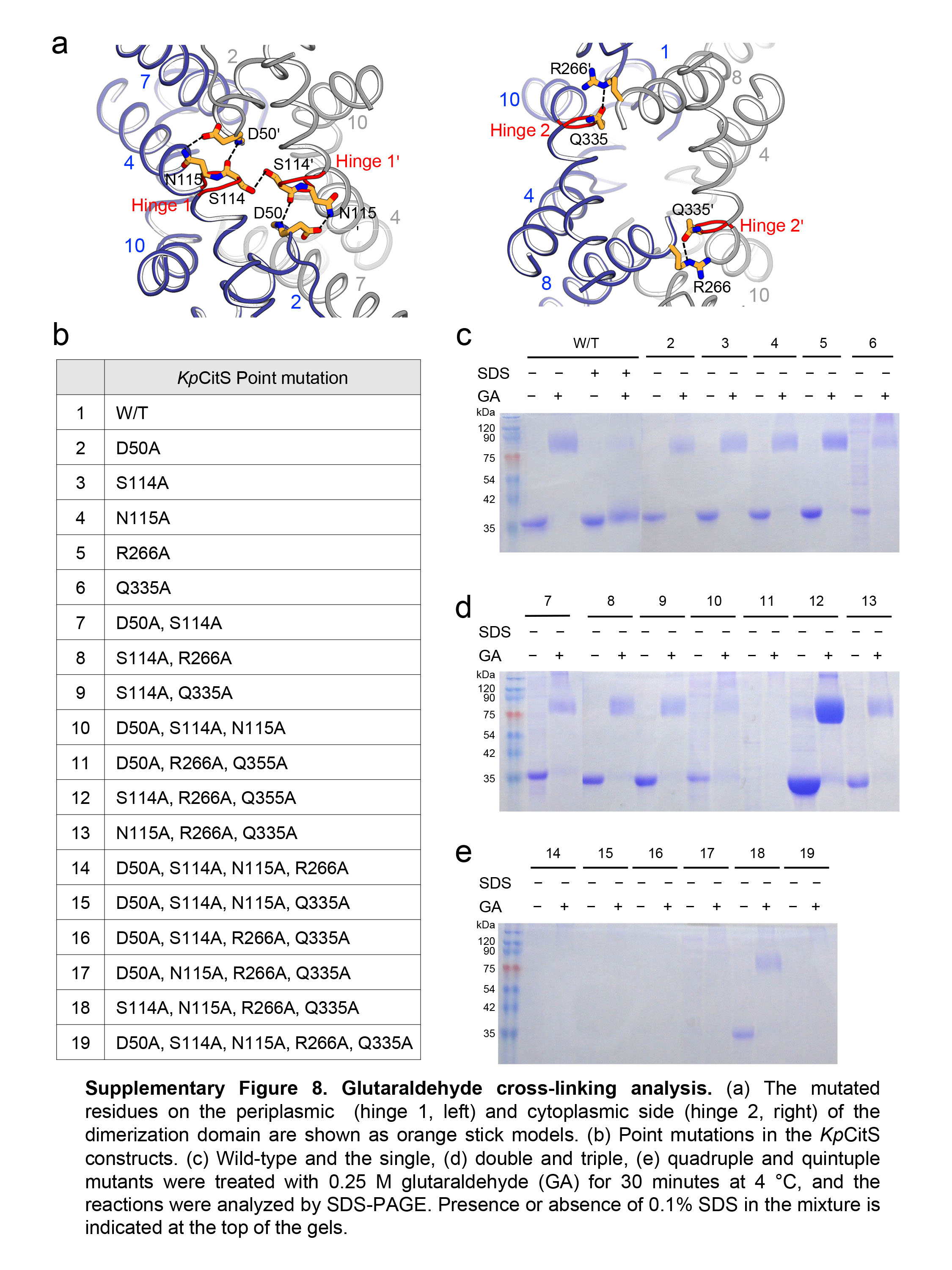
**

**Supplementary Figure 8. Glutaraldehyde cross-linking analysis.** (a) The mutated residues on the periplasmic (hinge 1, left) and cytoplasmic side (hinge 2, right) of the dimerization domain are shown as orange stick models. (b) Point mutations in the *Kp*CitS constructs. (c) Wild-type and the single, (d) double and triple, (e) quadruple and quintuple mutants were treated with 0.25 M glutaraldehyde (GA) for 30 minutes at 4 °C, and the reactions were analyzed by SDS-PAGE. Presence or absence of 0.1% SDS in the mixture is indicated at the top of the gels.

**
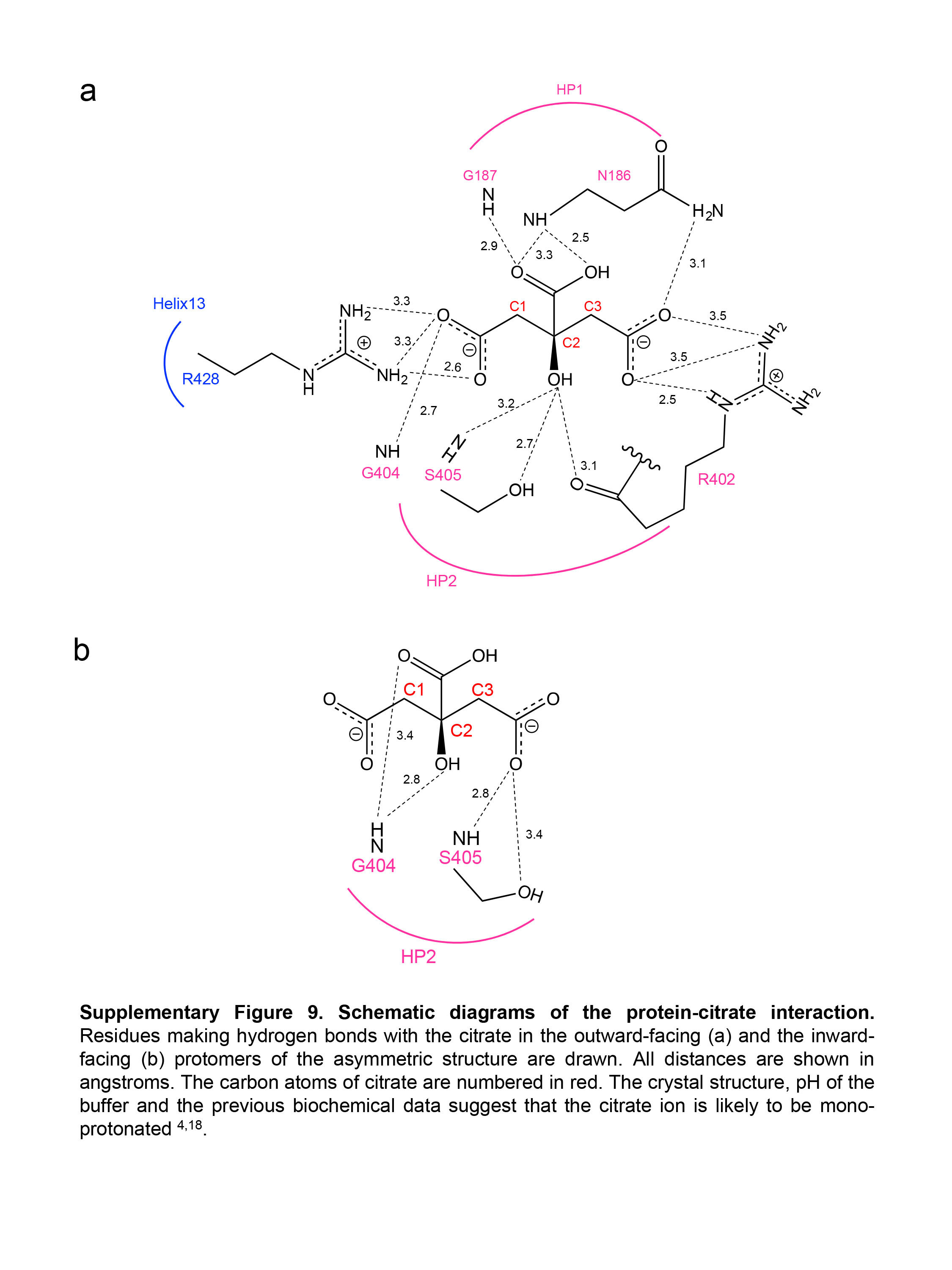
**

**Supplementary Figure 9. Schematic diagrams of the protein-citrate interaction.** Residues making hydrogen bonds with the citrate in the outward-facing (a) and the inward-facing (b) protomers of the asymmetric structure are drawn. All distances are shown in angstroms. The carbon atoms of citrate are numbered in red. The crystal structure, pH of the buffer and the previous biochemical data suggest that the citrate ion is likely to be mono-protonated^4,18^.

**
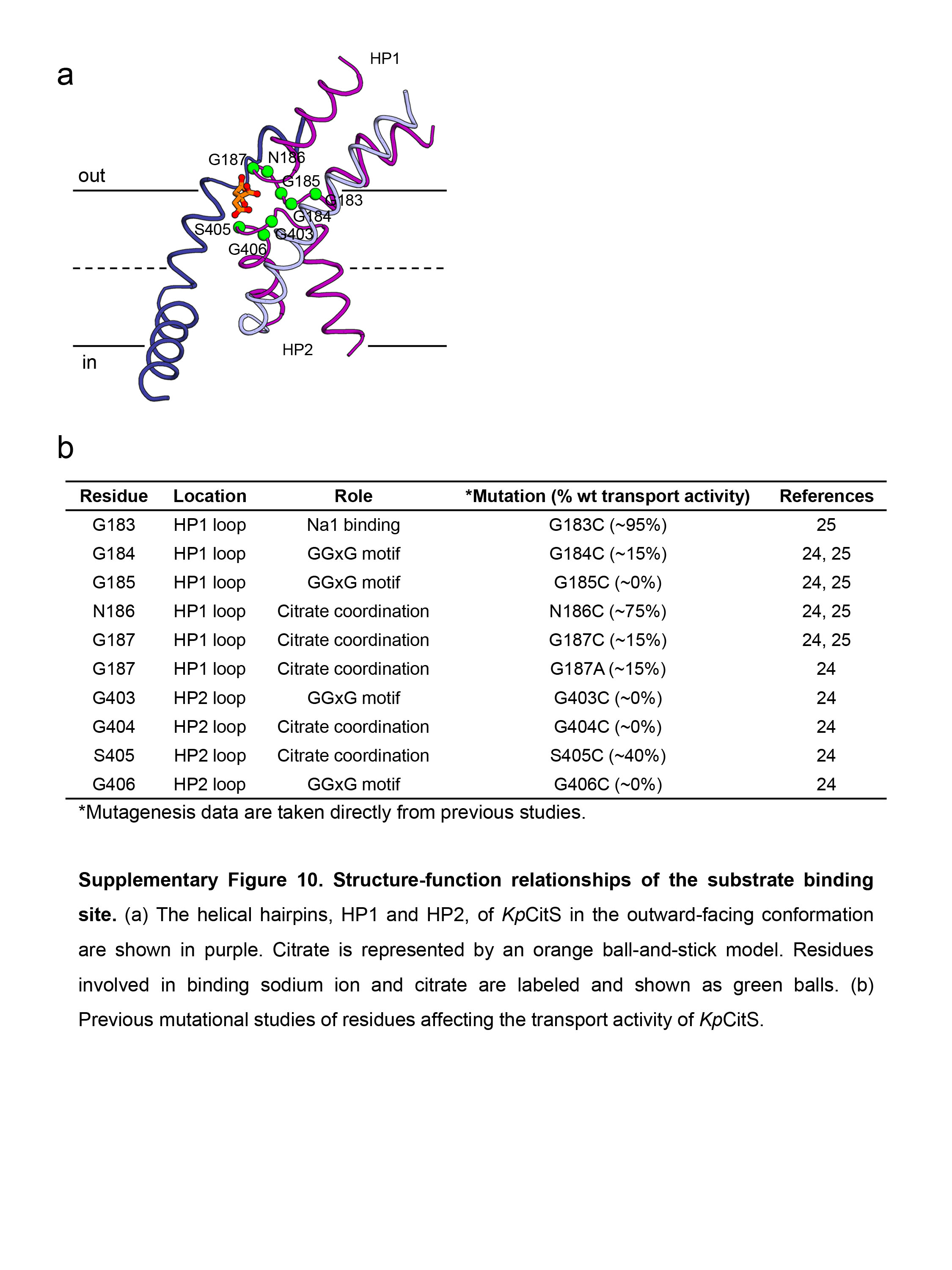
**

**Supplementary Figure 10. Structure-function relationships of the substrate binding site.** (a) The helical hairpins, HP1 and HP2, of *Kp*CitS in the outward-facing conformation are shown in purple. Citrate is represented by an orange ball-and-stick model. Residues involved in binding sodium ion and citrate are labeled and shown as green balls. (b) Previous mutational studies of residues affecting the transport activity of *Kp*CitS.

**
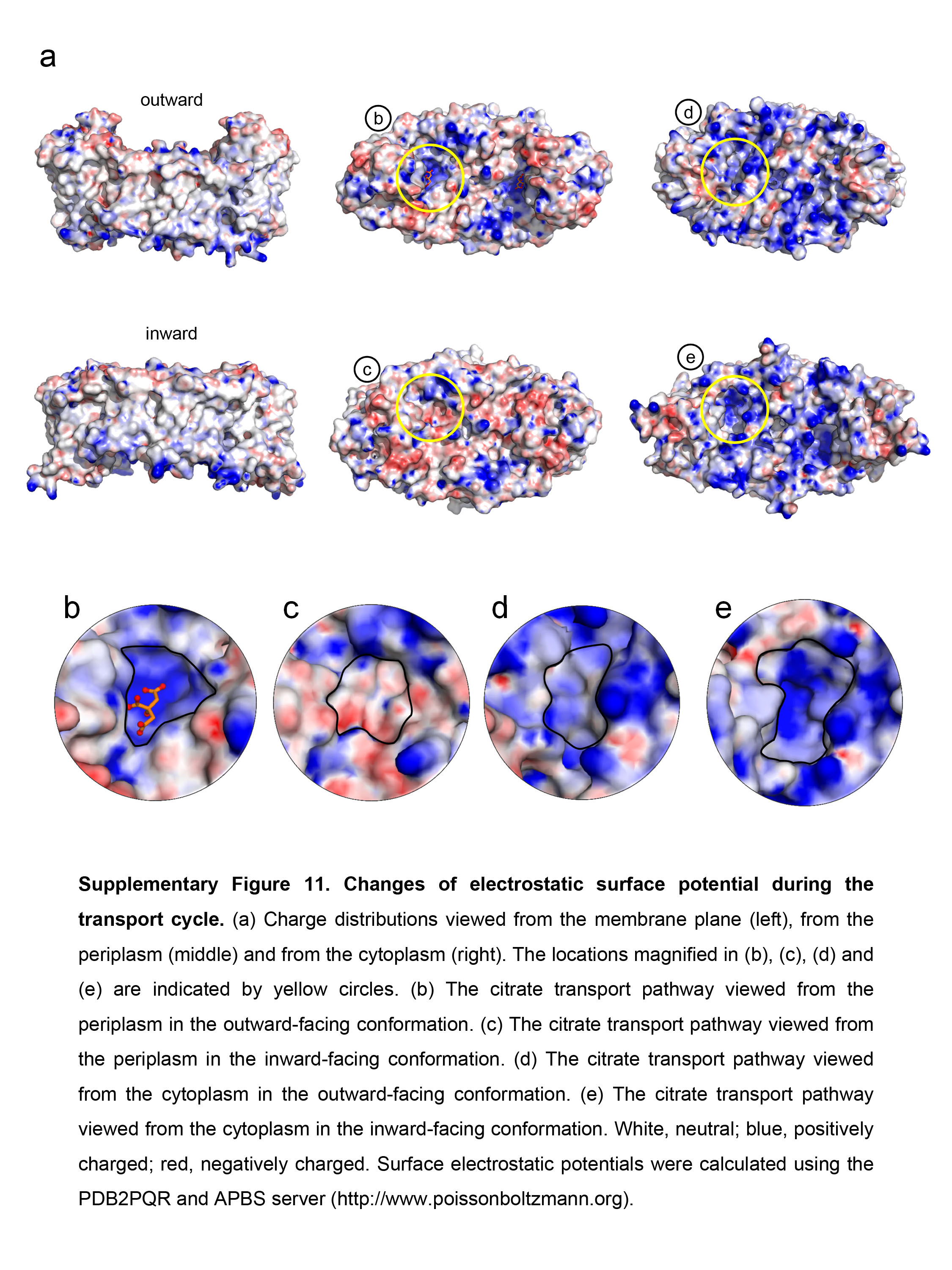
**

**Supplementary Figure 11. Changes of electrostatic surface potential during the transport cycle.** (a) Charge distributions viewed from the membrane plane (left), from the periplasm (middle) and from the cytoplasm (right). The locations magnified in (b), (c), (d) and (e) are indicated by yellow circles. (b) The citrate transport pathway viewed from the periplasm in the outward-facing conformation. (c) The citrate transport pathway viewed from the periplasm in the inward-facing conformation. (d) The citrate transport pathway viewed from the cytoplasm in the outward-facing conformation. (e) The citrate transport pathway viewed from the cytoplasm in the inward-facing conformation. White, neutral; blue, positively charged; red, negatively charged. Surface electrostatic potentials were calculated using the PDB2PQR and APBS server (http://www.poissonboltzmann.org).

**Supplementary Table 1.** Data collection and refinement statistics

| **Crystal form** | | 1 | | 2** | | 3 | | 4 |
| --- | --- | --- | --- | --- | --- | --- | --- | --- |
|  | Outward  (5X9R) | | Outward/Asymmetric  (5XAT) | | Inward  (5XAR) | | Asymmetric  (5XAS) | |
| **Data collection** |  | |  | |  | |  | |
| Space group | P4_1_ | | P1 | | P2_1_ | | P1 | |
| Cell dimensions |  | |  | |  | |  | |
| *a*, *b*, *c* (Å) | 93.9, 93.9, 172.9 | | 87.6, 91.8, 96.3 | | 90.3, 163.9, 93.6 | | 51.7, 83.3, 81.0 | |
|      *α, β, γ* (°) | 90.0, 90.0, 90.0 | | 86.0, 82.2, 69.6 | | 90.0, 93.5, 90.0 | | 97.1, 100.1, 94.5 | |
| Resolution (Å) | 50.0-4.0 (4.1-4.0)* | | 50.0-3.8 (3.9-3.8)* | | 50.0-3.6 (3.7-3.6)* | | 50.0-3.5 (3.6-3.5)* | |
| *R*_pim_ | 0.04 (0.58)* | | 0.04 (0.64)* | | 0.04 (0.76)* | | 0.07 (0.60)* | |
| I/σI | 30.2 (1.9)* | | 21.2 (1.5)* | | 22.7 (1.3)* | | 14.0 (1.5)* | |
| Completeness (%) | 99.2 (99.9)* | | 96.9 (98.6)* | | 99.6 (100.0)* | | 97.0 (94.8)* | |
| Redundancy | 6.8 (6.7)* | | 1.9 (1.9)* | | 3.7 (3.7)* | | 1.9 (1.8)* | |
|  |  | |  | |  | |  | |
| **Refinement** |  | |  | |  | |  | |
| Resolution (Å) | 50.0-4.0 | | 50.0-3.8 | | 50.0-3.6 | | 50-3.5 | |
| No. reflections (work/test) | 12147/596 | | 25473/1401 | | 29044/1433 | | 15446/849 | |
| *R*_work_ / *R*_free_ | 27.2/32.3 | | 26.7/29.8 | | 23.4/26.7 | | 24.5/29.5 | |
| No. atoms |  | |  | |  | |  | |
| Protein  Ligand | 6181  26 | | 12275  40 | | 12369  - | | 6109  26 | |
| Detergent  Ion | 25  - | | 21  3 | | 40  4 | | 20  2 | |
| Water | - | | 5 | | 11 | | 5 | |
| *B* factors |  | |  | |  | |  | |
| Protein  Ligand | 271.3  251.0 | | 240.7  218.0 | | 190.0  - | | 148.7  156.9 | |
| Detergent  Ion | 255.7  - | | 210.9  297.8 | | 221.9  194.0 | | 152.6  174.6 | |
| Water | - | | 219.9 | | 140.7 | | 126.1 | |
| R.m.s. deviations |  | |  | |  | |  | |
| Bond lengths (Å) | 0.008 | | 0.010 | | 0.016 | | 0.015 | |
| Bond angles (°)  Ramachandran plot (%)  Most favored  Allowed  Generously allowed  Disallowed | 1.24  92.2  7.5  0.3  0.0 | | 1.50  92.6  7.2  0.2  0.0 | | 1.74  94.7  5.3  0.0  0.0 | | 1.75  93.7  6.2  0.1  0.0 | |

*Numbers in parentheses were calculated with data in the highest resolution shell.

**Crystal form 2 contains the outward-facing and the asymmetric conformation of *Kp*CitS in the asymmetric unit.

Supplementary Table 2. CC1/2 of datasets

| **Crystal form 1** | | **Crystal form 2** | |
| --- | --- | --- | --- |
| **Outward** | | **Outward/Asymmetric*** | |
| **Resolution range (Å)** | **CC1/2** | **Resolution range (Å)** | **CC1/2** |
| 50.00-8.60 | 0.99 | 50.00-8.18 | 0.99 |
| 8.60-6.84 | 0.99 | 8.18-6.49 | 0.99 |
| 6.84-5.97 | 0.98 | 6.49-5.67 | 0.99 |
| 5.97-5.43 | 0.96 | 5.67-5.16 | 0.99 |
| 5.43-5.04 | 0.96 | 5.16-4.79 | 0.99 |
| 5.04-4.74 | 0.95 | 4.79-4.50 | 0.99 |
| 4.74-4.50 | 0.92 | 4.50-4.28 | 0.97 |
| 4.50-4.31 | 0.87 | 4.28-4.09 | 0.90 |
| 4.31-4.14 | 0.63 | 4.09-3.94 | 0.84 |
| 4.14-4.00 | 0.64 | 3.94-3.80 | 0.78 |

| **Crystal form 3** | | **Crystal form 4** | |
| --- | --- | --- | --- |
| **Inward** | | **Asymmetric** | |
| **Resolution range (Å)** | **CC1/2** | **Resolution range (Å)** | **CC1/2** |
| 50.00-7.75 | 0.99 | 50.00-7.53 | 0.99 |
| 7.75-6.15 | 0.99 | 7.53-5.98 | 0.99 |
| 6.15-5.38 | 0.98 | 5.98-5.23 | 0.97 |
| 5.38-4.89 | 0.98 | 5.23-4.75 | 0.98 |
| 4.89-4.54 | 0.98 | 4.75-4.41 | 0.97 |
| 4.54-4.27 | 0.97 | 4.41-4.15 | 0.94 |
| 4.27-4.05 | 0.93 | 4.15-3.94 | 0.87 |
| 4.05-3.88 | 0.87 | 3.94-3.77 | 0.76 |
| 3.88-3.73 | 0.69 | 3.77-3.63 | 0.99 |
| 3.73-3.60 | 0.53 | 3.63-3.50 | 0.54 |

*Crystal form 2 contains the outward-facing and the asymmetric conformation of *Kp*CitS in the asymmetric unit.

**References**

56 McCoy, A. J. *et al.* Phaser crystallographic software. *J Appl Crystallogr* **40**, 658-674, doi:10.1107/S0021889807021206 (2007).

57 Emsley, P. & Cowtan, K. Coot: model-building tools for molecular graphics. *Acta Crystallogr D Biol Crystallogr* **60**, 2126-2132, doi:10.1107/S0907444904019158 (2004).

58 Brunger, A. T. Version 1.2 of the Crystallography and NMR system. *Nat Protoc* **2**, 2728-2733, doi:10.1038/nprot.2007.406 (2007).

59 Murshudov, G. N., Vagin, A. A. & Dodson, E. J. Refinement of macromolecular structures by the maximum-likelihood method. *Acta Crystallogr D Biol Crystallogr* **53**, 240-255, doi:10.1107/S0907444996012255 (1997).

60 Adams, P. D. *et al.* PHENIX: a comprehensive Python-based system for macromolecular structure solution. *Acta Crystallogr D Biol Crystallogr* **66**, 213-221, doi:10.1107/S0907444909052925 (2010).

61 Sali, A. & Blundell, T. L. Comparative protein modelling by satisfaction of spatial restraints. *J Mol Biol* **234**, 779-815, doi:10.1006/jmbi.1993.1626 (1993).
